# Emodin Alleviates Sepsis‐Induced Multiorgan Damage by Inhibiting NETosis through Targeting Neutrophils BCL‐10

**DOI:** 10.1002/advs.202417129

**Published:** 2025-08-08

**Authors:** Xiaolong Xu, Yumeng Yan, Meiling Zheng, Mina Zhang, Tengfei Chen, Zhicheng Qu, Yinglu Bai, Shuwen Zhang, Chunming Zhao, Yifan Shi, Yan Lin, Ning Wang, Yunjing Bai, Yating Zhai, Zhaofang Bai, Wei Guo, Qingquan Liu

**Affiliations:** ^1^ Beijing Hospital of Traditional Chinese Medicine Capital Medical University Beijing Institute of Chinese Medicine Beijing Key Laboratory of Basic Research with Traditional Chinese Medicine on Infectious Diseases Beijing 100010 China; ^2^ School of Traditional Chinese Medicine Beijing University of Chinese Medicine Beijing 102488 China; ^3^ Dongzhimen Hospital Beijing University of Chinese Medicine Beijing 100007 China; ^4^ Department of Hepatology The Fifth Medical Center Chinese PLA General Hospital Beijing 100039 China

**Keywords:** BCL‐10/MALT1, Emodin, NETosis, NF‐κB, sepsis

## Abstract

Sepsis is a life‐threatening condition caused by dysregulated host responses to infection, characterized by excessive inflammation and abnormal coagulation. Neutrophil extracellular traps (NETs) formation bridges these two pathological processes. Through both in vivo and in vitro experiments, it is observed that Emodin, a natural anthraquinone derivative derived from Dahuang, significantly ameliorates the cytokine storm and coagulation abnormalities induced by sepsis, demonstrating remarkable efficacy in inhibiting NETs formation. Furthermore, through protein microarrays, surface plasmon resonance (SPR), pull‐down assays, and molecular docking analyses, BCL‐10 is established as a direct target of Emodin, providing protective effects in both in vivo and in vitro settings. Through conditional knockout of BCL‐10 in neutrophils, alongside single‐cell sequencing analyses, it is confirmed that BCL‐10 is key in promoting excessive NET formation in sepsis. Additionally, Emodin exerts powerful protective effects by modulating the function of the BCL‐10/MALT1 complex, thereby alleviating the NF‐κB signaling activation and inhibiting NETs formation. Collectively, these findings provide pharmacological evidence that Emodin targeted BCL‐10 regulates the BCL‐10/MALT1 complex and suppresses NF‐κB activation, ultimately conferring significant multiorgan protective effects in sepsis. The conduct of this study provides new clues for the translational research of Emodin and its target BCL‐10 in sepsis.

## Graphical abstract

Emodin targets BCL‐10 to modulate the BCL‐10/MALT1 complex, thereby suppressing NF‐κB activation and significantly exerting multiorgan protective effects in sepsis.

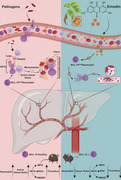

## 1 Introduction

Sepsis is a life‐threatening organ dysfunction caused by a dysregulated host response to infection and is a severe complication of critical conditions such as infection, burns, trauma, and shock.^[^ ^1^ ^]^ Epidemiological studies estimate that there are approximately 31.5 million cases of sepsis and 19.4 million cases of severe sepsis globally each year.^[^ ^2^ ^]^ The mortality rate of sepsis is reported to be between 30% and 50%, with septic shock patients experiencing mortality rates as high as 60% to 80%.^[^ ^3^ ^]^ Traditionally, the overwhelming infection and systemic inflammatory response have been considered the primary pathological mechanisms of sepsis.^[^ ^4^ ^]^ However, clinical studies have shown that anti‐inflammatory treatment alone does not significantly improve sepsis survival rates, suggesting that sepsis involves more complex pathophysiological mechanism.^[^ ^5^ ^]^ Therefore, understanding the underlying mechanisms of sepsis and developing effective therapeutic interventions remain critical challenges.

As research into sepsis has advanced, excessive inflammatory activation and coagulation dysfunction have been recognized as core mechanisms driving disease progression. The hallmark of early sepsis is a dysregulated systemic inflammatory response to infection. Upon pathogen entry, pathogen‐associated molecular patterns (PAMPs) are recognized by pattern recognition receptors on monocytes and neutrophils, including Toll‐like receptors (TLRs), NOD‐like receptors (NLRs), and RIG‐I‐like receptors (RLRs).^[^ ^1^ ^]^ This recognition activates neutrophils, leading to the release of proinflammatory cytokines such as tumor Necrosis Factor‐alpha (TNF‐α), interleukin‐1(IL‐1), interleukin‐6(IL‐6), and interferon‐gamma(IFN‐γ)^[^ ^6^ ^]^ underscoring the central role of neutrophils in the inflammatory response during sepsis. When the infection is not effectively controlled, damage‐associated molecular patterns (DAMPs), such as high‐mobility group box 1 (HMGB1), are released, further amplifying the inflammatory response and contributing to immune dysregulation.^[^ ^7^ ^]^ Clinical studies indicate that ≈25% to 50% of sepsis patients develop disseminated intravascular coagulation (DIC), a pathological condition closely associated with poor prognosis.^[^ ^8^ ^]^ In the later stages of sepsis, the excessive immune response not only triggers a cytokine storm but also activates the coagulation pathway. HMGB1 induces tissue factor expression on monocytes, enhancing procoagulant activity and promoting platelet aggregation, ultimately leading to the formation of immune thrombosis.^[^ ^9^ ^]^ Thus, the interplay between inflammation and coagulation is significant in the pathophysiology of sepsis. Identifying key targets and mechanisms to inhibit the excessive activation of both processes is crucial for the treatment of sepsis.

In the excessive activation of inflammation and coagulation, neutrophil extracellular traps (NETs) play a critical role. NETs are extracellular fibrous networks released by activated neutrophils, primarily composed of DNA, granular proteins, and antimicrobial enzymes, designed to capture pathogens.^[^ ^10^ ^]^ However, excessive NETosis can exacerbate the inflammatory response, acting as a “coconspirator” in the pathogenesis of sepsis. Recent studies have revealed that B‐cell lymphoma 10 (BCL‐10), a critical adaptor protein of the caspase recruitment domain (CARD) family, plays a pivotal regulatory role in innate immunity through the formation of the CBM signaling complex (CARD9/BCL‐10/MALT1).^[^ ^11^ ^]^ On the one hand, BCL‐10 orchestrates the recognition of PAMPs and DAMPs, thereby modulating innate immune responses and inducing the production of inflammatory cytokines. On the other hand, MALT1, a key downstream signaling mediator of the CBM complex, drives the cascade amplification of NF‐κB signaling via its dual functions as a scaffold and a proteolytic enzyme. This mechanism not only reinforces the formation of a proinflammatory positive feedback loop but also directly promoting the release of NETs. During inflammation, platelets interact with neutrophils via Toll‐like receptor 4 (TLR4), promoting both NETosis and thrombosis.^[^ ^12^, ^13^ ^]^ Additionally, NETs activate endothelial cells (ECs), damage the vascular barrier, and enhance neutrophil transendothelial migration, further promoting the formation of NETs.^[^ ^14^, ^15^, ^16^ ^]^ This process establishes NETs as key mediators of the hyperactivation of inflammation and coagulation in sepsis. Therefore, effectively inhibiting NET formation through targeting the BCL‐10/NF‐κB axis may provide a critical therapeutic strategy for alleviating coagulation dysfunction in sepsis.

Emodin is a potent anthraquinone bioactive compound primarily derived from the traditional Chinese medicine rhubarb (Dahuang). Research has demonstrated that Emodin possesses significant anti‐inflammatory, antioxidant, and organ‐protective effects.^[^ ^17^ ^]^ It alleviates lipopolysaccharide (LPS)‐induced acute lung injury by inhibiting the activity of the NOD‐, LRR‐ and pyrin domain‐containing protein 3 inflammasome.^[^ ^18^ ^]^ Furthermore, Emodin significantly suppresses the inflammatory response in cecal ligation and puncture (CLP)‐induced sepsis, reducing intestinal mucosal damage.^[^ ^19^, ^20^ ^]^ Additionally, research suggests that Emodin can improve hypercoagulability by modulating the activation of neutrophil N2 phenotypes.^[^ ^21^ ^]^ The phenotypic heterogeneity of neutrophils plays a pivotal role in the regulation of inflammatory responses. Depending on microenvironmental signals, neutrophils can polarize into a proinflammatory N1 phenotype or an immunosuppressive N2 phenotype. Recent studies have demonstrated that neutrophils in both septic and recovery phases exhibit immunosuppressive properties.^[^ ^22^ ^]^ In the septic microenvironment, persistent PAMPs and DAMPs drive the polarization of neutrophils toward the N2 phenotype. These N2‐polarized neutrophils are characterized by high expression of arginase‐1 and cluster of differentiation 206, which promote the formation of an immunosuppressive pathological state through mechanisms such as upregulation of immune checkpoint molecules (e.g., PD‐L1) to suppress T‐cell function. Despite these encouraging findings, most current studies focus on evaluating therapeutic outcomes and lack in‐depth investigation into the molecular targets and mechanisms. Notably, the role of Emodin in regulating inflammation‐coagulation dysfunction and NETs formation in CLP‐induced sepsis has yet to be reported. This gap presents a critical opportunity for further research to uncover Emodin's full potential in addressing these pathological processes.

In this study, we explored the effects of Emodin on NETs formation and evaluated its protective role in a CLP‐induced sepsis mouse model. Our findings indicate that sepsis is characterized by severe inflammation–coagulation dysregulation, with NETs playing a central role in mediating these pathological processes. Emodin significantly inhibited the formation of NETs, thereby disrupting the excessive activation of the inflammation and coagulation systems. Furthermore, we found that Emodin exerts its protective effects in sepsis by targeting and inhibiting BCL‐10, which modulates nuclear factor kappa‐light‐chain‐enhancer of activated B cells (NF‐κB) activity, thus providing a crucial mechanism for sepsis intervention.

## 2 Results

### 2.1 Emodin Protected against Mortality and Multiorgan Pathological Damage in Sepsis Mice

**Figure 1:**
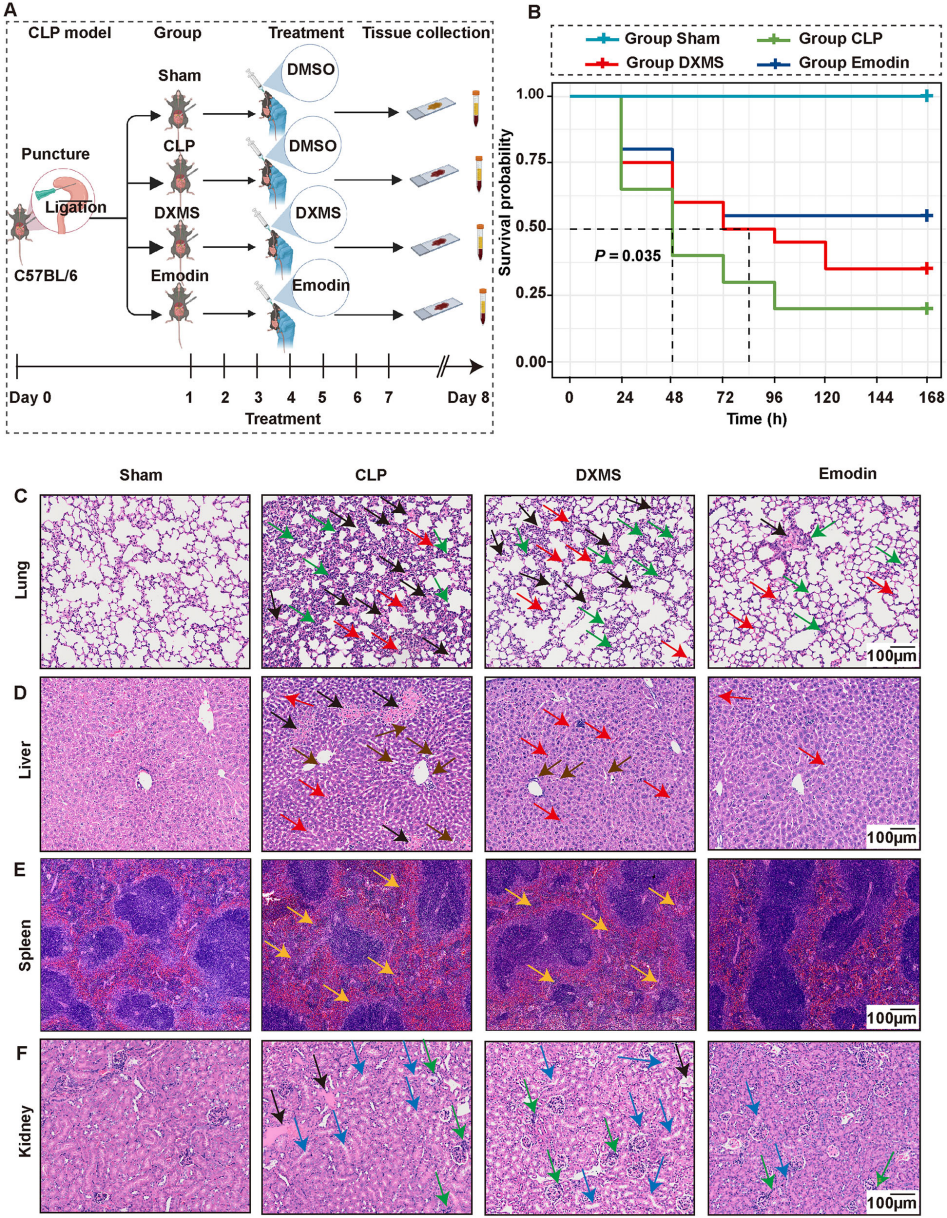
Emodin alleviated mortality and multiorgan injury in CLP‐induced septic mice. A) Schematic overview of the experimental design, including the modeling, group allocation, and intervention timeline. B) Kaplan–Meier survival curves over a 7‐day period comparing the Sham, CLP septic group, Emodin treatment group, and DXMS treatment group. C–E) Histopathological images showing tissue morphology and injury in the lung (C), liver (D), spleen (E), and kidney (F) across the four experimental groups.

To investigate the pharmacological effects of Emodin on mortality and multiorgan damage in sepsis, a CLP‐induced sepsis mouse model was established. Mice were randomly assigned to four groups: Sham, model, Emodin intervention, and dexamethasone (DXMS) treatment groups (**Figure** **1**A). As shown in Figure 1B, analysis of 7‐day survival rates demonstrated statistically significant differences among these groups (*P* = 0.035). Both the model and DXMS groups exhibited significantly lower survival rates compared to the Sham group, particularly at 48 and 72 h. In contrast, the Emodin intervention group showed a markedly higher survival rate, suggesting that Emodin effectively reduced mortality in septic mice, highlighting its potential protective role. Subsequent hematoxylin and eosin (H&E) staining of multiorgan tissues indicated that Emodin treatment significantly improved pathological outcomes in the lung, liver, spleen, and kidneys. The CLP sepsis group exhibited severe alveolar destruction characterized by diffuse inflammatory infiltration (green arrows), microthrombosis (black arrows), capillary hemorrhage, and alveolar septal thickening (red arrows). While DXMS partially reduced inflammatory infiltration, Emodin significantly alleviated these pathological changes (Figure 1C). Hepatic analysis revealed disrupted lobular architecture, hepatocyte steatosis (brown arrows), vascular rupture with erythrocyte extravasation (red arrows), and widespread microthrombi (black arrows) in CLP mice. DXMS failed to resolve erythrocyte leakage or steatotic vacuolation, whereas Emodin only showed localized hepatocyte swelling and sporadic vascular damage (Figure 1D). Histopathological analysis of the spleen revealed a significant increase in spleen weight in the CLP‐induced sepsis model group (Figure S1, Supporting Information). Histological examination further demonstrated structural disorganization of the red and white pulp, manifested as blurred boundaries and hyperplastic fusion of the white pulp regions (yellow arrows). Notably, Emodin intervention effectively reversed these pathological alterations, restoring distinct demarcation between red and white pulp and significantly suppressing white pulp hyperplasia (Figure 1E). Renal pathology in CLP mice manifested tubular epithelial edema (blue arrows), interstitial inflammatory infiltration (green arrows), and microthrombosis (black arrows). DXMS exacerbated cellular edema, while Emodin preserved renal structural integrity with markedly reduced interstitial hemorrhage and inflammation (Figure 1F). Collectively, these multiorgan findings demonstrated that Emodin significantly reduced multiorgan failure risk, with organ‐protective efficacy surpassing DXMS monotherapy.

### 2.2 Emodin Mitigated Systemic Inflammatory Dysregulation and Pathological Coagulation Activation Induced by Sepsis

**Figure 2:**
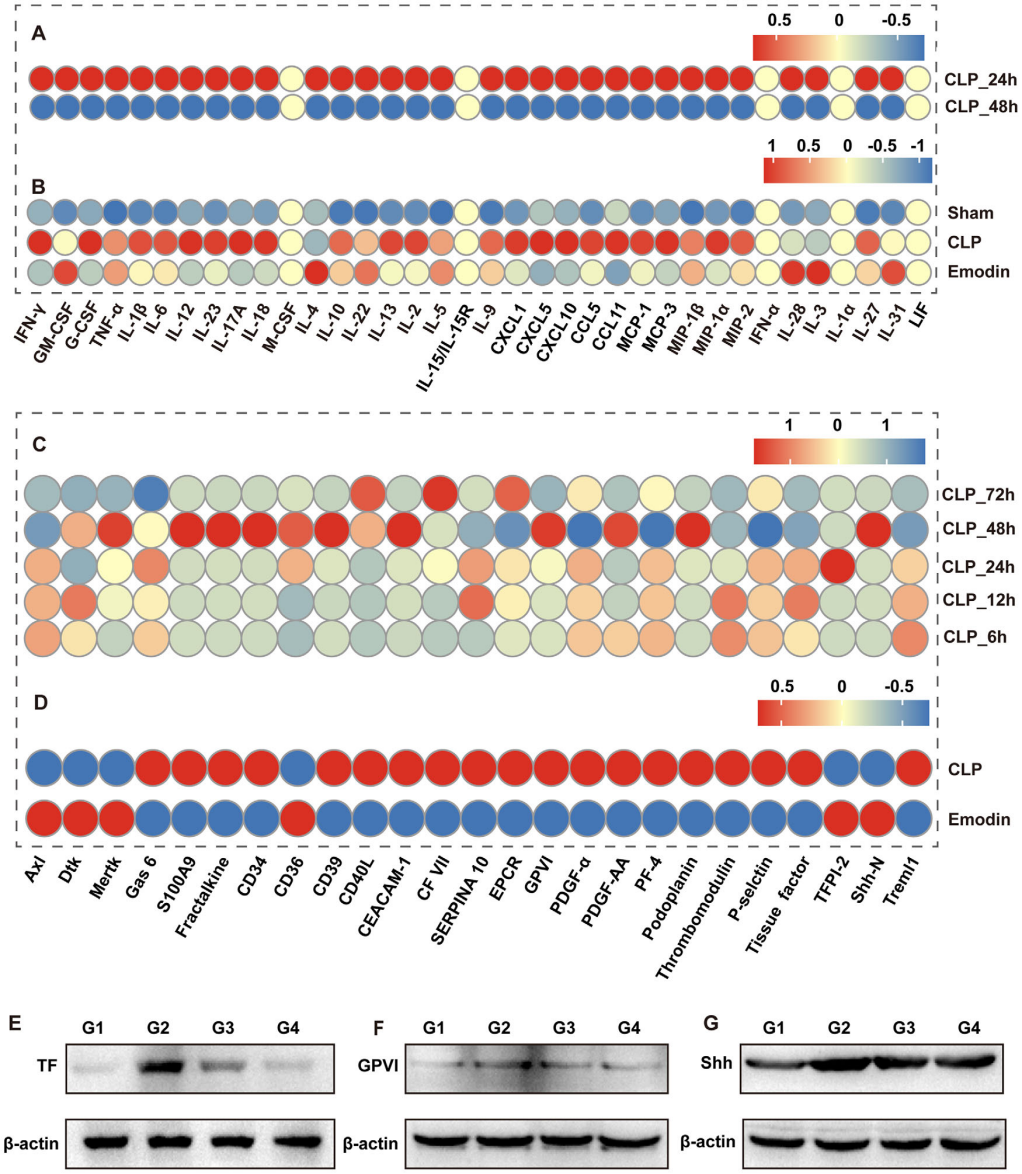
Emodin‐reduced inflammatory response and coagulation abnormalities in CLP‐induced septic mice. A) Expression levels of various inflammatory cytokines and chemokines in the peripheral plasma of CLP‐induced septic mice at 24 and 48 h postsurgery. B) Comparative expression levels of inflammatory cytokines and chemokines in the peripheral plasma between the CLP‐induced septic group and the Emodin treatment group at 24 h postsurgery. C) Time course analysis of multiple coagulation‐related factors in peripheral plasma of CLP‐induced septic mice at 6, 12, 24, 48, and 72 h postsurgery. E,F) WB analysis of protein expression levels for TF (E), GPVI (F), and Shh (G) in liver tissues of the four experimental groups at 48 h postsurgery. G1, G2, G3, and G4 represent the Sham group, CLP model group, Emodin intervention group, and DXMS intervention group, respectively.

Using a multifactor chip assay, we analyzed the expression of inflammation‐ and coagulation‐related factors in the peripheral plasma of septic mice at various time points. Results indicated a significant upregulation of multiple inflammatory factors at 24 h postsepsis, followed by a decline at 48 h (**Figure** **2**A). Concurrently, coagulation factor activation rose significantly, peaking at 48 h (Figure 2C). Notably, Emodin intervention significantly suppressed inflammatory factor expression at 24 h (Figure 2B), with a corresponding reduction in coagulation factor levels observed at 48 h (Figure 2D). Additionally, we used western blotting (WB) analysis to assess tissue factor (TF), glycoprotein VI (GPVI) and sonic hedgehog (Shh) protein levels in liver tissues, as these proteins are closely linked to coagulation cascade activation. Compared to the sham group, septic mice showed significant upregulation of TF, GPVI, and Shh, which was markedly downregulated by Emodin treatment (Figure 2E–G). These findings suggest that sepsis induces a time‐dependent systemic dysregulation of inflammation and abnormal coagulation activation. Emodin intervention helps modulate this dysregulation, promoting a return to homeostatic balance.

### 2.3 Emodin‐Inhibited NETs Formation in Septic Mice

**Figure 3:**
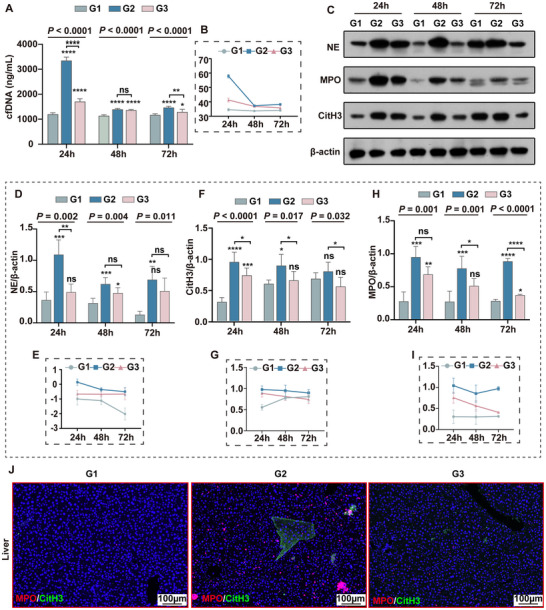
Emodin inhibited the formation of NETs in CLP‐induced septic mice. A) Bar graph showing the expression levels of cfDNA in different groups of mice at various time points (24, 48, and 72 h). B) Line graph illustrating the expression levels of cfDNA in different groups of mice at 24, 48, and 72 h. C) WB analysis of NE, MPO, and CitH3 protein expression in liver tissues of different groups of mice at various time points (24, 48, and 72 h). D,E) Bar graph (D) and line graph (E) showing the expression levels of NE protein in liver tissues of mice at different time points (24, 48, and 72 h). F,G) Bar graph (F) and line graph (G) depicting the expression levels of CitH3 protein in liver tissues of mice at different time points (24, 48, and 72 h). H,I) Bar graph (H) and line graph (I) represent the expression levels of MPO protein in liver tissues of mice at different time points (24, 48, and 72 h). J) Multiplex IF images of liver tissues from different groups stained for MPO and CitH3. G1, G2, and G3 represent the sham group, CLP model group, and Emodin intervention group, respectively. *P < 0.05, **P < 0.01, ***P < 0.001, ****P < 0.0001, n.s., *P* > 0.05 (no significant difference).

Given the potential role of NETs in driving excessive inflammation and coagulation activation during sepsis, we assessed key NETs components in septic mice, including cell‐free DNA (cfDNA), myeloperoxidase (MPO), neutrophil elastase (NE) and citrullinated histone H3 (CitH3). Plasma cfDNA levels were measured at 24, 48, and 72 h post‐CLP induction, showing significant group, time, and group–time interaction effects (Table S1, Supporting Information). cfDNA levels revealed notable differences among groups at each time point (*P* < 0.0001) (**Figure** **3**A). At 24 h, cfDNA levels were markedly higher in the model group than in the Sham group (*P* < 0.0001), while Emodin treatment significantly reduced cfDNA levels (*P* < 0.0001). Additionally, cfDNA levels declined at 48 and 72 h in both the model and Emodin‐treated groups (*P* < 0.0001) (Figure 3B).

We further analyzed NE, MPO, and CitH3 protein levels in liver tissues via WB. NE expression exhibited significant group, time, and group–time interaction effects (Table S2, Supporting Information). Specifically, NE levels varied significantly among groups at 24 h (*P* = 0.002), 48 h (*P* = 0.004), and 72 h (*P* = 0.011) (Figure 3C,D). Compared to Sham, the CLP group had elevated NE levels at all time points (*P* < 0.01), with Emodin treatment significantly reducing NE expression at 24 h (*P* < 0.01), but not at later times. In the CLP group, NE levels decreased from 24 to 48 h (*P* = 0.009) and further at 72 h (*P* = 0.005) (Figure 3E).

For CitH3 expression, group and group–time interaction effects were significant, though time effects were not (Table S3, Supporting Information). Intergroup differences were observed at 24 h (*P* < 0.0001), 48 h (*P* = 0.017), and 72 h (*P* = 0.032) (Figure 3C,F). CitH3 levels rose significantly in the CLP group at 24 h (*P* < 0.0001) and 48 h (*P* < 0.05), with Emodin treatment significantly lowering CitH3 levels at each time point (*P* < 0.05) and showing a progressive decline (*P* = 0.045) (Figure 3G).

MPO levels showed significant group effects (*P* < 0.0001) and group–time interactions (*P* < 0.0001), without notable time effects (*P* = 0.123) (Table S4, Supporting Information). Significant differences among groups appeared at 24 h (*P* = 0.001), 48 h (*P* = 0.001), and 72 h (*P* < 0.0001) (Figure 3C–H). MPO expression was higher in the CLP group across all time points (*P* < 0.001), while Emodin treatment significantly lowered MPO at 48 h (*P* < 0.05) and 72 h (*P* < 0.0001). In the Emodin‐treated group, MPO expression progressively decreased over time (*P* < 0.001) (Figure 3I).

We further evaluated the effect of Emodin on sepsis‐induced hepatic NETs using multiplex immunofluorescence (IF) techniques. The results showed that, compared to the Sham group, the number of MPO and CitH3 positive cells and their fluorescence intensities significantly increased in the sepsis group. Emodin treatment significantly reduced both the number of positive cells and fluorescence intensities (Figure 3J). These findings indicated that the CLP‐induced sepsis model promotes extensive NETs formation, and that Emodin intervention can effectively reduce NETs formation, thereby mitigating inflammatory damage.

### 2.4 Emodin‐Inhibited Neutrophil NETs Formation

**Figure 4:**
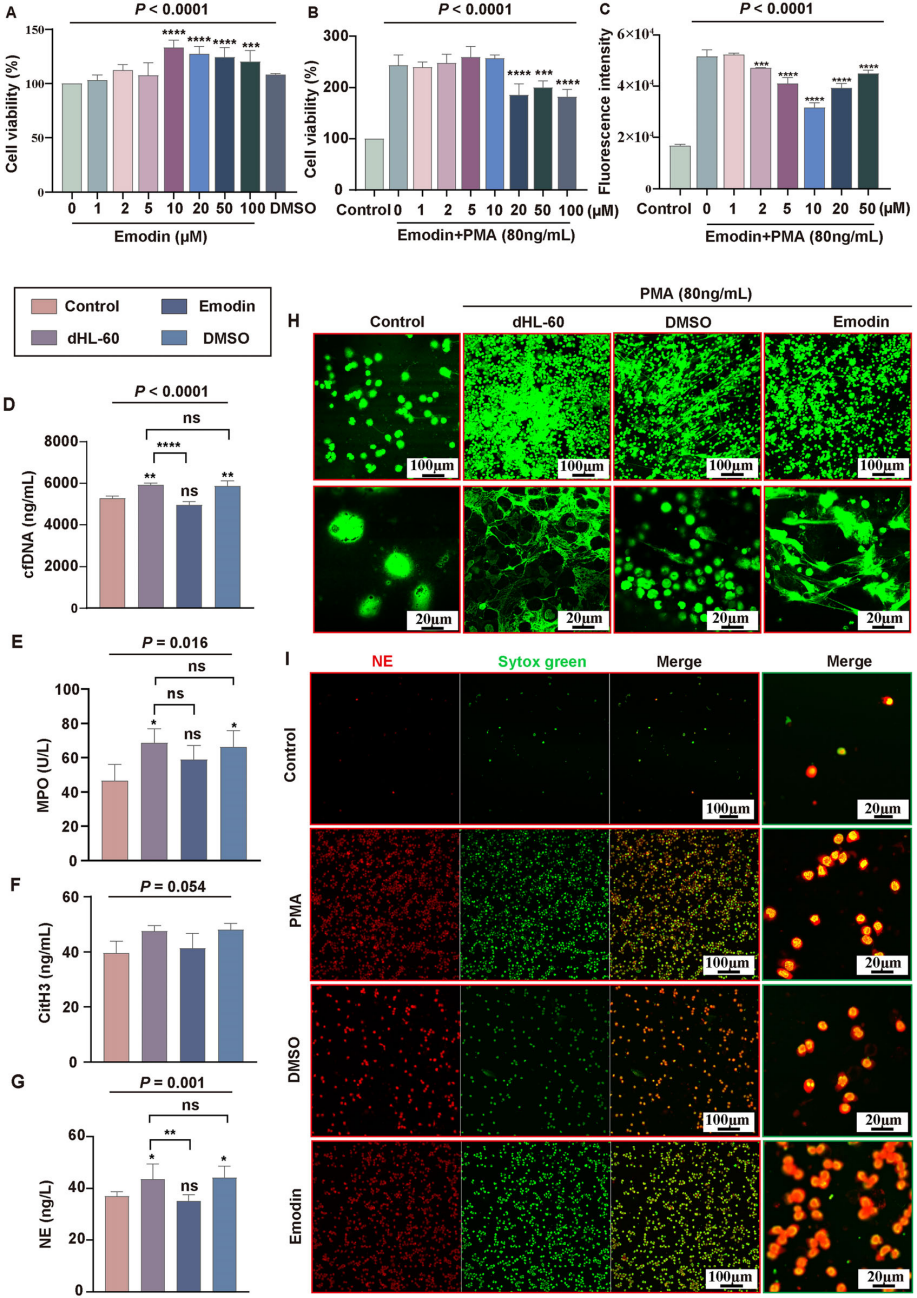
Emodin significantly inhibited NETs formation in vitro. A) CCK‐8 assay to evaluate the effect of Emodin on the viability of dHL‐60 cells. B) CCK‐8 assay assessing the impact of Emodin on dHL‐60 cells after PMA induction. C) Dichlorodihydrofluorescein diacetate (DCFH‐DA) probe detection of ROS activity in dHL‐60 cells after PMA induction, with or without Emodin treatment. D) PicoGreen fluorescence assay measuring cfDNA levels in cell supernatants across different groups. E) MPO content was measured using a colorimetric MPO assay kit in various groups of cells. F,G) Enzyme‐linked immunosorbent assay (ELISA) quantification of NE (F) and CitH3 (G) levels in the cell supernatants of different groups. H) Sytox Green fluorescence staining of cells across different experimental groups. I) Immunofluorescence staining detecting NE protein expression in the various groups of cells. *P < 0.05, **P < 0.01, ***P < 0.001, ****P < 0.0001, n.s., *P* > 0.05 (no significant difference).

To examine Emodin's effect on NETs formation in vitro, Human acute promyelocytic leukemia cell Line‐60 (HL‐60) cells were differentiated into neutrophil‐like cells using all‐trans retinoic acid (ATRA). Wright–Giemsa staining confirmed that the optimal ATRA concentration was 2 µm with a 96 h induction period (Figure S2, Supporting Information). The optimal phorbol 12‐myristate 13‐acetate (PMA) concentration for inducing NETs formation was determined to be 80 ng mL^−1^ with a 3 h stimulation, based on reactive oxygen species (ROS) production in dHL‐60 cells (Figure S3, Supporting Information). Cell counting kit‐8 (CCK‐8) assays indicated no significant cytotoxicity of Emodin on dHL‐60 cells (**Figure** **4**A), although Emodin notably reduced cell viability under PMA stimulation (Figure 4B). Furthermore, ROS assays showed that Emodin significantly decreased ROS production in PMA‐stimulated dHL‐60 cells, with the strongest effect observed at 10 µm, which was chosen for further experiments (Figure 4C). These results suggest that Emodin has the potential to inhibit neutrophil activation. To evaluate Emodin's impact on NETs formation, levels of NETs‐related components, including cfDNA, MPO, NE, and CitH3, were measured in cell supernatants. PMA stimulation significantly increased cfDNA levels compared to controls (*P* < 0.01), while Emodin treatment markedly reduced cfDNA expression (*P* < 0.01) (Figure 4D). NE levels followed a similar pattern, with significant group differences (*P* = 0.001), and Emodin significantly inhibited PMA‐induced NE upregulation (*P* < 0.01) (Figure 4G). Although MPO levels showed group differences, the reduction in MPO expression in the Emodin group compared to PMA alone was not statistically significant but displayed a downward trend (Figure 4E). CitH3 levels did not show statistically significant differences (*P* = 0.054) (Figure 4F).

Sytox Green staining further demonstrated that while control cells remained round without NETs formation, PMA stimulation induced nuclear deformation, yielding a net‐like, filamentous structure indicative of NETs. Emodin treatment significantly reduced NETs structures, confirming its inhibitory effect on NETs formation (Figure 4H). Immunofluorescence (IF) staining of NE showed that PMA upregulated NE expression, while Emodin treatment reduced NE levels (Figure 4I). In summary, Emodin displayed significant potential in inhibiting neutrophil activity and NETs formation in vitro.

### 2.5 BCL‐10 Was Pivotal in Emodin‐Mediated Inhibition of NETs Formation

**Figure 5:**
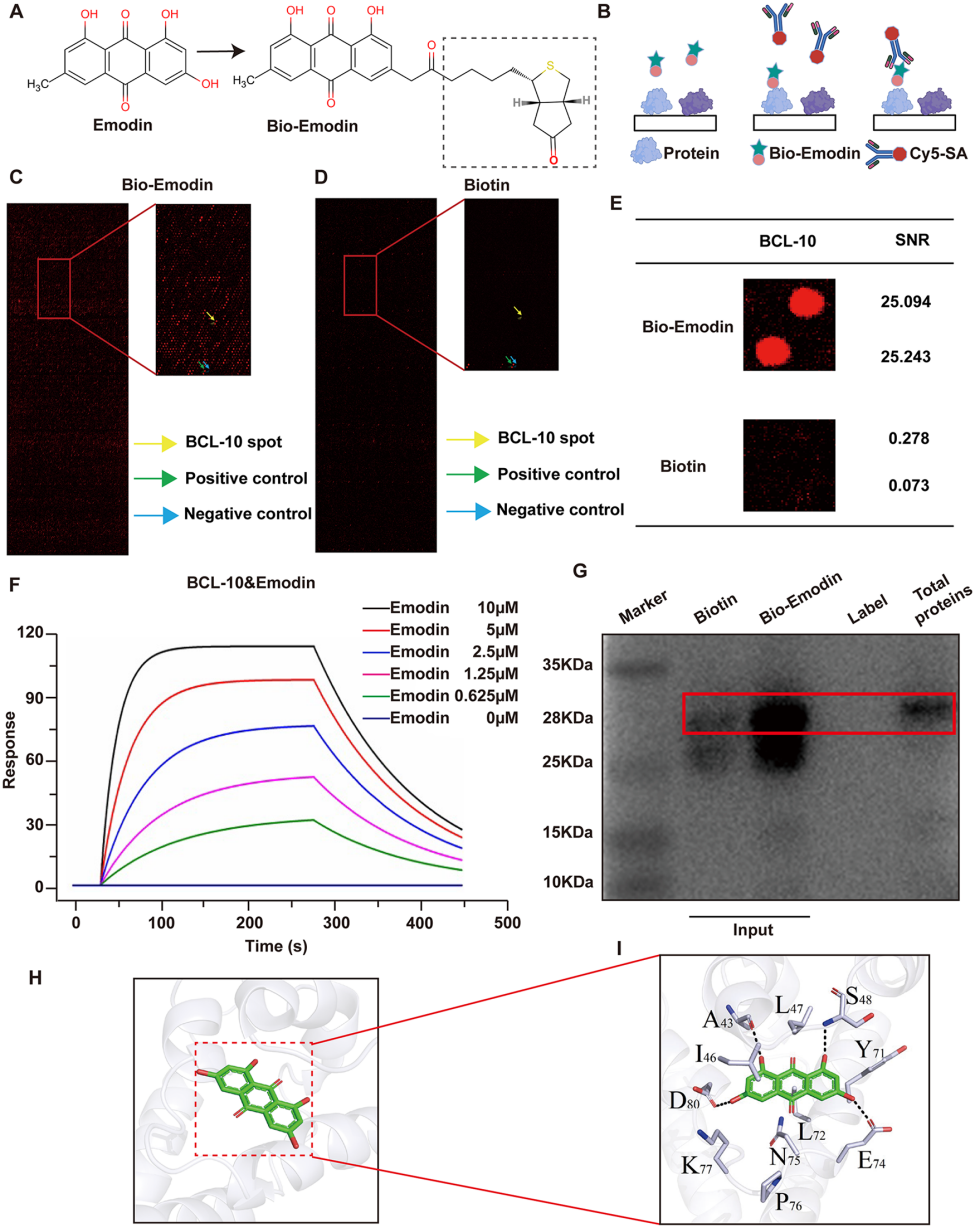
Identification of BCL‐10 as a binding protein of Emodin. A) Chemical structures of Emodin and biotin‐labeled Emodin (Bio‐Emodin). B) Schematic representation of the steps involved in identifying Emodin‐binding proteins using a recombinant human protein microarray. C,D) Representative images of the protein array showing positive control (green arrows) and negative control (blue arrows) spots, as well as the spot corresponding to BCL‐10 (yellow arrow). E) An enlarged image displaying the binding of Bio‐Emodin to the BCL‐10 spot on the protein array, indicating SNR. F) SPRi fitting curve for the interaction between Emodin and BCL‐10. G. Pull‐down experiments showing the interaction of BCL‐10 protein with Emodin in liver tissues from CLP sepsis mice. H,I) Molecular docking images of Emodin with BCL‐10, including the structural diagram of the lowest binding energy conformation (H) and a representation of the key interacting residues (I).

To investigate the molecular mechanism by which Emodin inhibits sepsis‐induced NETs formation, we screened for Emodin‐binding proteins. Using biotin‐labeled Emodin (Bio‐Emodin) (**Figure** **5**A), we assessed interactions with recombinant proteins on the HuProt human protein microarray. After incubation with Bio‐Emodin or free biotin, binding was detected via Cy5‐conjugated streptavidin (Cy5‐SA) (Figure 5B–D). The results identified BCL‐10 as a key candidate protein with a strong binding potential to Emodin, demonstrated by a signal‐to‐noise ratio (SNR) of 25.168 (Figure 5E).

To further confirm this interaction, we performed surface plasmon resonance (SPR) and molecular docking studies. SPR analysis showed that Emodin binds to rhBCL‐10 with a KD of 1.94 × 10^−⁶^ m, indicating a high binding affinity (Figure 5F). Additionally, a biotinylated protein interaction pull‐down assay validated Emodin's binding to BCL‐10 in mouse liver tissue lysates. Bio‐Emodin was immobilized on streptavidin agarose beads and incubated with liver lysates, confirming Bio‐Emodin's binding to BCL‐10 in this complex mixture (Figure 5G). Further molecular docking and simulation studies, using the BCL‐10 crystal structure (PDB: 6BEZ), revealed that Emodin interacts with BCL‐10′s binding pocket with an optimal docking energy of −7.59 kcal mol^−1^ (Figure 5H). Molecular dynamics simulations with AMBER14 software suggested that Emodin's hydrophobic planar groups engage in stable hydrophobic interactions with BCL‐10′s hydrophobic residues, while hydroxyl (─OH) and carbonyl (═O) groups form strong hydrogen bonds with hydrophilic residues in the binding pocket. The molecular dynamics estimated the binding energy at −7.03 kcal mol^−1^, supporting a high binding affinity (Figure 5I).

To rigorously validate the direct and specific binding between emodin and BCL‐10, we have performed the following functional experiments as recommended: pull‐down assay, competitive inhibition assay, and thermal shift assay (Figure S5, Supporting Information). For the pull‐down assay, emodin was covalently conjugated to Sepharose beads (Emodin–Sepharose) as bait. Cell lysates overexpressing Flag‐BCL‐10 were incubated with either Emodin–Sepharose or Control–Sepharose (drug‐free beads). Western blot analysis using an anti‐Flag antibody clearly demonstrated that Flag‐BCL‐10 was significantly pulled down by Emodin–Sepharose but not by Control–Sepharose (Figure S5A, Supporting Information). This result confirms the specific and direct interaction between Emodin and BCL‐10 in cell lysates, effectively excluding artifacts from the beads themselves or nonspecific protein binding. Furthermore, to test whether free Emodin competes for the same binding site on BCL‐10, lysates containing Flag‐BCL‐10 were preincubated with Emodin–Sepharose followed by addition of free Emodin (100 µm). Results showed that free Emodin competitively inhibited the binding of Flag‐BCL‐10 to Emodin–Sepharose (Figure S5B, Supporting Information), demonstrating that Emodin targets a defined binding pocket on BCL‐10 where free and conjugated drugs compete for the identical site. Finally, target engagement and conformational stabilization were validated through thermal shift assay. Recombinant BCL‐10 protein was incubated with either DMSO (solvent control) or 100 µm Emodin, followed by gradient thermal treatment (37 °C, 42 °C, 47 °C, 52 °C, 57 °C, 62 °C, 67 °C). Key results revealed that at high temperatures (62 °C and 67 °C), the Emodin‐treated group maintained detectable soluble BCL‐10 protein signals, whereas BCL‐10 in the DMSO control group was almost completely denatured and precipitated with markedly diminished or absent signals (Figure S5C, Supporting Information). This indicates that Emodin binding significantly enhances BCL‐10′s thermal resistance, preserving its solubility and structural integrity at elevated temperatures, and directly demonstrates that Emodin binding stabilizes the native conformation of BCL‐10. In conclusion, these findings suggest that BCL‐10 is a primary target of Emodin and may play a crucial role in mediating Emodin's inhibition of NETs formation.

### 2.6 Emodin‐Mediated Targeting of Neutrophil BCL‐10 Suppresses NETs Formation in Sepsis

**Figure 6:**
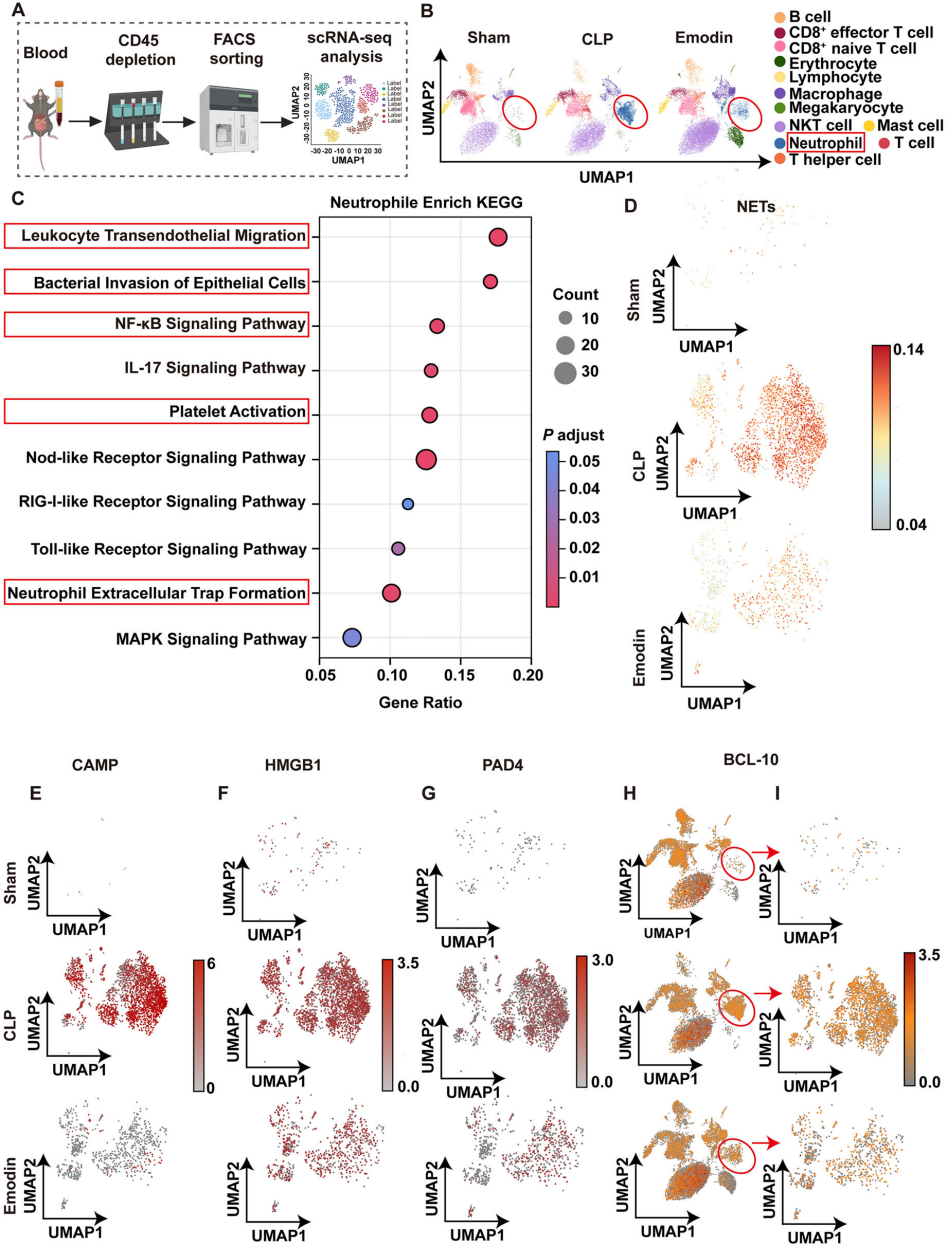
Single‐cell sequencing showed that Emodin targets BCL‐10 to inhibit NETs formation. A) Schematic diagram of the single‐cell sequencing process. B) Visualization of high‐dimensional data of whole blood samples from different experimental groups (sham group, CLP model group, and Emodin intervention group) using UMAP technology. C) KEGG analysis displaying the enrichment signaling pathways of upregulated genes in the CLP model group. D) UMAP plots of the NETs signaling pathway in the sham group, CLP model group, and Emodin intervention group. E–G) UMAP plots showing the distribution of CAMP (E), HMGB1 (F), and PAD4 (G) in the Sham group, CLP model group, and Emodin intervention group. H,I) UMAP visualization of the distribution and enrichment of BCL‐10 in all cells (H) and neutrophils (I).

To further explore the roles of various cell populations in sepsis, we performed transcriptomic sequencing on the peripheral blood of septic mice (**Figure** **6**A). The immune cell populations in the sham, CLP, and Emodin intervention groups included B cells, CD8^+^ effector T cells, CD8^+^ naïve T cells, erythrocytes, lymphocytes, macrophages, megakaryocytes, natural killer T cells, mast cells, neutrophils, T cells, and T helper cells (Figure 6B). The uniform manifold approximation and projection (UMAP) plot demonstrated a significant enrichment of neutrophils in the CLP group compared to the sham group, with a notable reduction following Emodin treatment. Next, we conducted Kyoto encyclopedia of genes and genomes (KEGG) pathway enrichment analysis on the top differentially expressed genes within neutrophils from septic mice. As shown in Figure 6C, pathways related to leukocyte transendothelial migration, bacterial invasion of epithelial cells, inflammation (including the NF‐κB signaling pathway), NETs formation, and platelet activation were significantly enriched, indicating an inflammatory, NETs‐forming, and coagulation‐activated state in septic neutrophils. UMAP visualization of NETs enrichment showed a marked increase in the CLP group compared to the Sham group, which was significantly reduced by Emodin intervention (Figure 6D). Key NETs‐related genes, such as cyclic adenosine monophosphate (CAMP), HMGB1, and peptidyl arginine deiminase 4 (PAD4) (Figure 6E–G), exhibited similar trends.

Further analysis of BCL‐10, a target protein of Emodin, revealed broad expression across various cell types, with notable enrichment in neutrophils. Specifically, BCL‐10 expression was significantly elevated in CLP group neutrophils and reduced following Emodin treatment (Figure 6H,I). Correlation analysis between BCL‐10 and key NETs‐related genes, including CAMP, HMGB1, and PAD4, showed a positive correlation with statistical significance (Figure S4, Supporting Information). In summary, these results suggest that neutrophil hyperactivation, NETs formation, and high BCL‐10 expression are central to sepsis pathogenesis. BCL‐10 may serve as a regulatory gene promoting NETs formation, with Emodin potentially mitigating sepsis by targeting BCL‐10 and inhibiting NETs formation.

### 2.7 BCL‐10 Deficiency Improved Septic Phenotypes and NETs Formation in Mice

**Figure 7:**
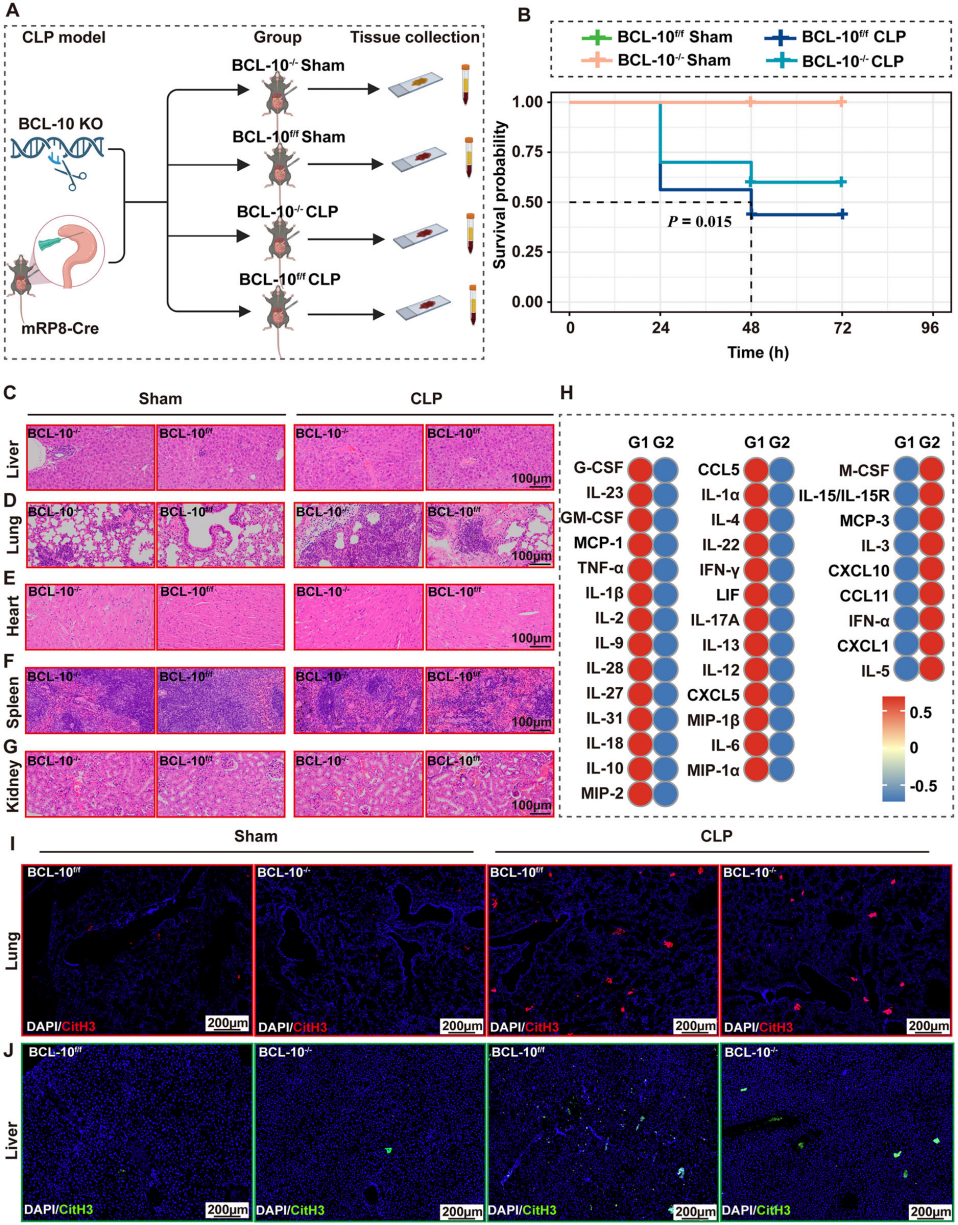
The absence of BCL‐10 alleviated organ damage and NETs formation induced by CLP sepsis. A) Schematic representation of the construction, modeling, and grouping of BCL‐10^f/f^ and BCL‐10^−/−^ mice. B) Kaplan–Meier survival curves over a 7‐day period comparing the survival rates among BCL‐10^f/f^ CLP, BCL‐10^f/f^ sham, BCL‐10^−/−^ CLP, and BCL‐10^−/−^ sham groups. The survival curves of the BCL‐10^f/f^ sham group and BCL‐10^−/−^ sham group exhibit complete overlap due to 100% survival rates in both cohorts. C‐G. Histopathological images of the liver, lungs, heart, spleen, and kidneys from BCL‐10^f/f^ CLP, BCL‐10^f/f^ sham, BCL‐10^−/−^ CLP, and BCL‐10^−/−^ sham groups. H) Expression levels of multiple inflammatory cytokines and chemokines in BCL‐10^f/f^ CLP and BCL‐10^−/−^ CLP groups. I,J) IF images showing CitH3 in the liver (I) and lung (J) tissues of BCL‐10^f/f^ CLP, BCL‐10^f/f^ sham, BCL‐10^−/−^ CLP, and BCL‐10^−/−^ sham groups. G1, and G2 represent the BCL‐10^−/−^ CLP group and BCL‐10^f/f^ CLP group.

To further investigate the role of BCL‐10 in sepsis, we crossed BCL‐10^flox/flox^ mice with mRP8‐Cre mice to generate a neutrophil‐specific BCL‐10 knockout model: mRP8‐Cre BCL‐10^flox/flox^ (BCL‐10^−/−^) mice and BCL‐10^flox/flox^ (BCL‐10^f/f^) mice, for studying the function of BCL‐10 in sepsis (**Figure** **7**A). The results showed that, compared to BCL‐10f/f CLP mice, BCL‐10^−/−^ CLP mice exhibited significantly reduced mortality (*P* = 0.015) (Figure 7B). Additionally, BCL‐10^−/−^ CLP mice demonstrated significant amelioration of multiple organ pathologies, including reduced inflammatory cell infiltration in the portal vein and hepatic steatosis (Figure 7C), as well as decreased inflammatory infiltration in the lungs and heart (Figure 7D,E), clearer separation of red and white pulp in the spleen (Figure 7F), and less tubular cell swelling in the kidneys (Figure 7G). Furthermore, BCL‐10^−/−^ CLP mice exhibited a significant reduction in the expression of several inflammatory cytokines and chemokines, such as TNF‐α, IL‐1β, C‐X‐C motif chemokine ligand 1 (CXCL1), and CXCL5, in peripheral plasma (Figure 7H), indicating a marked reduction in systemic inflammatory activation. IF analysis further revealed a significant decrease in NETs formation (CitH3) in the lung and liver tissues of BCL‐10^−/−^ CLP mice (Figure 7I,J). In conclusion, our study suggests that neutrophil‐specific deletion of BCL‐10 significantly alleviates CLP‐induced septic pathology and NETs formation.

### 2.8 Single‐Cell RNA Sequencing Revealed BCL‐10 Deficiency Reduces Neutrophil Infiltration and NETs Formation

**Figure 8:**
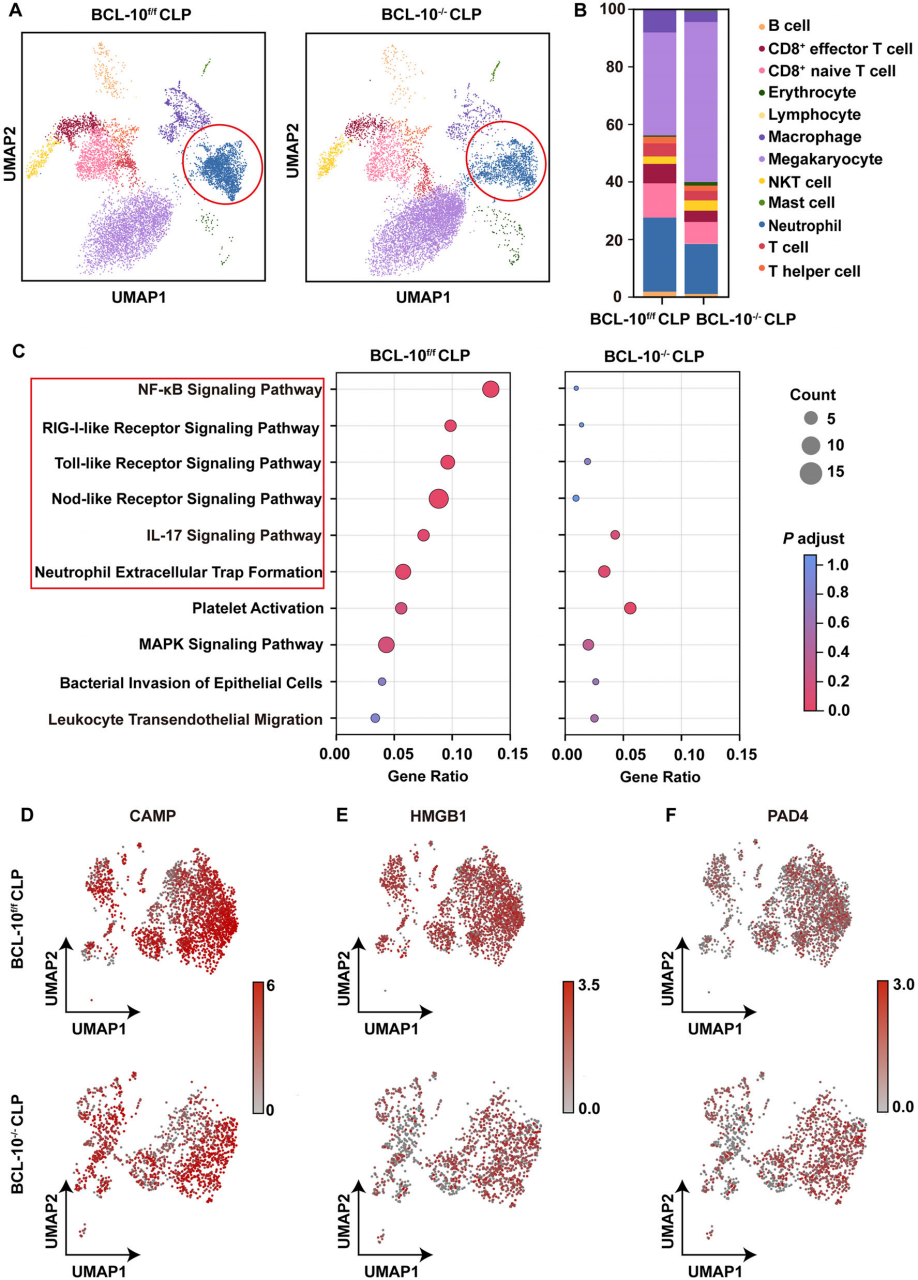
Single‐cell sequencing showed that the absence of BCL‐10 reduces neutrophil enrichment and NETs formation in CLP sepsis mice. A) UMAP visualization of whole blood samples from BCL‐10^f/f^ CLP and BCL‐10^−/−^ CLP mice. B) Bar graph showing the proportions of different cell types in the whole blood samples of BCL‐10^f/f^ CLP and BCL‐10^−/−^ CLP mice. C) KEGG analysis displaying the enrichment signaling pathways of upregulated genes in the BCL‐10^f/f^ CLP model group compared to the BCL‐10^−/−^ CLP group. D–F) UMAP plots showing the expression of CAMP (D), HMGB1 (E), and PAD4 (F) in the BCL‐10^f/f^ CLP and BCL‐10^−/−^ CLP groups.

Peripheral blood samples from BCL‐10^f/f^ and BCL‐10^−/−^ CLP mice were collected for single‐cell RNA sequencing to evaluate the impact of BCL‐10 deficiency on various cell populations in septic mice. Unbiased clustering analysis identified 12 distinct cell clusters, with distribution and proportions shown in **Figure** **8**A,B. Compared to BCL‐10^f/f^ CLP mice, BCL‐10^−/−^ CLP mice demonstrated a significant reduction in neutrophil enrichment and proportion. KEGG pathway enrichment analysis on the top upregulated genes in neutrophils from BCL‐10^f/f^ CLP mice, compared directly with BCL‐10^−/−^ CLP mice, revealed substantial suppression of several inflammatory pathways, including the NF‐κB signaling pathway, RLR signaling pathway, and NETs formation in BCL‐10^−/−^ CLP mice (Figure 8C). Key NETs‐related genes, such as CAMP, HMGB1, and PAD4, also showed significantly reduced expression (Figure 8D–F). In conclusion, these findings suggested that BCL‐10 deficiency suppressed neutrophil infiltration, activation, and NETs formation, thereby alleviating CLP‐induced septic pathology.

### 2.9 Emodin‐Inhibited NETs Formation by Regulating NF‐κB Activation through Modulation of BCL‐10‐MALT1 Function

**Figure 9:**
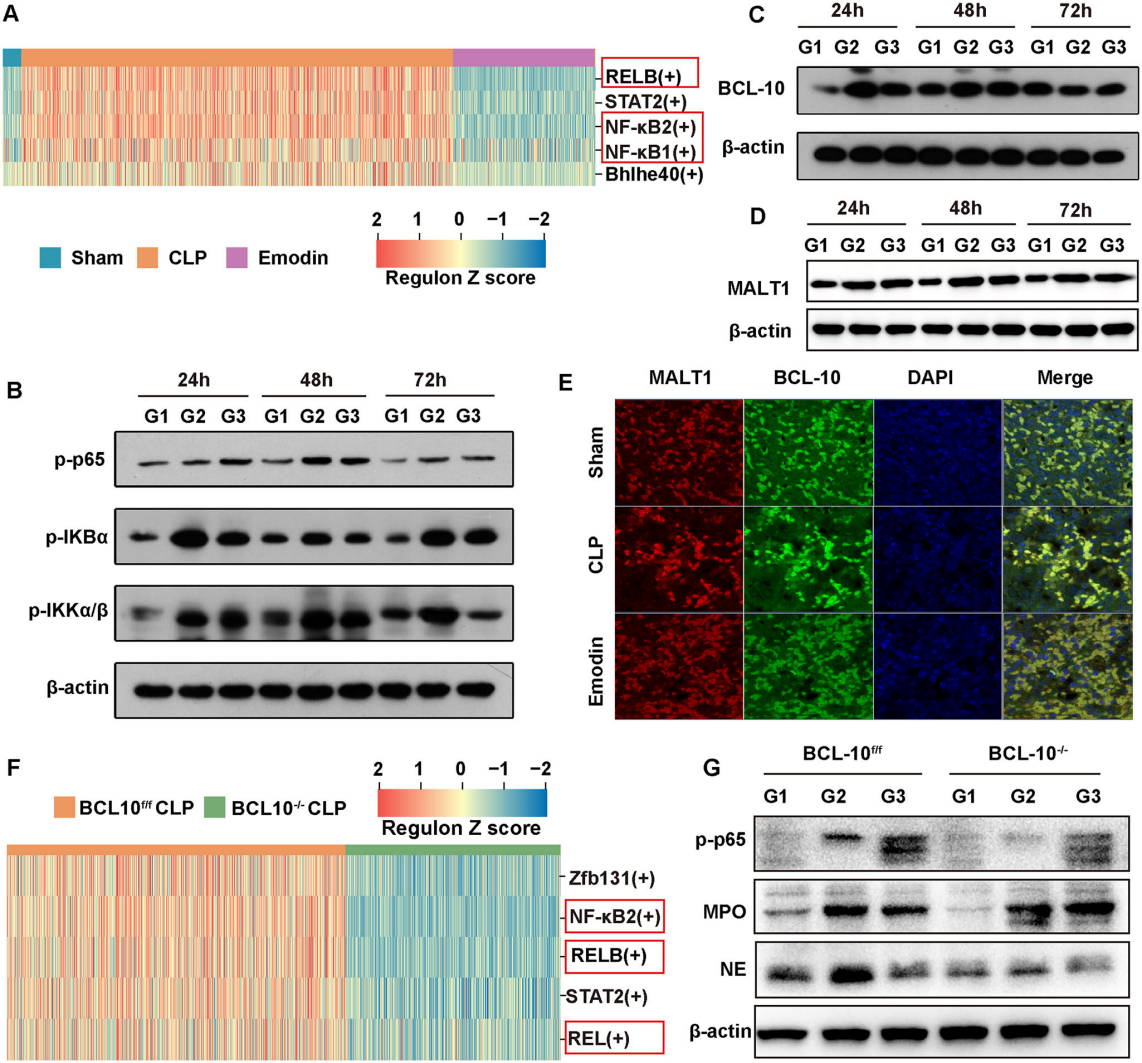
Emodin regulated NF‐κB activation and inhibits NETs formation by affecting the function of the BCL‐10/MALT1 complex. A) Heatmap of regulon activity in neutrophils from the Sham group, CLP model group, and Emodin intervention group, analyzed using SCENIC software. B) WB analysis of the phosphorylation levels of NF‐κB p65, IκBα, and IKKα/β in liver tissues from the Sham group, CLP model group, and Emodin intervention group at different time points (24 h/48 h/72 h). C,D) WB analysis of BCL‐10 protein and MALT1 expression levels in liver tissues from the Sham group, CLP model group, and Emodin intervention group at different time points (24 h/48 h/72 h). E) IF imaging showing the expression of the BCL‐10/MALT1 complex in liver tissues from different mouse groups. F) Heatmap of regulon activity in neutrophils from BCL‐10^f/f^ CLP and BCL‐10^−/−^ CLP groups, analyzed using SCENIC software. G) WB analysis of p‐p65, MPO, and NE expression levels in liver tissues from BCL‐10^f/f^ and BCL‐10^−/−^ mice across different groups. G1, G2, and G3 represent the sham group, CLP model group, and Emodin intervention group, respectively.

To explore the role of the BCL‐10‐MALT1 complex in sepsis, we analyzed proteins in its upstream and downstream NF‐κB signaling pathway. Single‐cell sequencing results indicated that inflammation‐ and immunity‐related transcription factors, including v‐rel reticuloendotheliosis viral oncogene homolog B (RELB), NF‐κB1, NF‐κB2, and signal transducer and activator of transcription 2 (STAT2), were significantly upregulated in septic mice compared to the Sham group. RELB, NF‐κB1, and NF‐κB2, crucial in both classical and nonclassical NF‐κB pathways, were elevated, suggesting NF‐κB pathway activation in sepsis. Emodin treatment reduced the activity of these transcription factors, indicating NF‐κB pathway inhibition (**Figure** **9**A). For NF‐κB p65, Table S5 of the Supporting Information shows significant intergroup differences (*P* < 0.0001). CLP upregulated p‐p65 expression (*P* < 0.0001), while Emodin treatment reduced it significantly (*P* < 0.0001) (Table S6, Supporting Information), especially at 24 and 48 h. Similar trends were observed in inhibitor of kappa B alpha (IKBα) and IκB kinase alpha/beta (IKKα/β) phosphorylation, with Emodin effectively suppressing their phosphorylation (Tables S7–S9, Supporting Information). These findings suggest that NF‐κB signaling is highly activated in sepsis, while Emodin has substantial inhibitory potential.

Multiplex immunofluorescence analysis showed elevated MALT1 and BCL‐10 expression in liver tissues of the CLP group, particularly BCL‐10, indicating NF‐κB pathway activation (Figure 9E). Emodin treatment reduced both MALT1 and BCL‐10 expressions, confirmed by WB analysis (*P* < 0.0001) (Figure 9C,D), especially significant at 24 and 48 h (*P* < 0.0001) (Tables S10 and S11, Supporting Information). This suggests that Emodin inhibits NETs formation by modulating the BCL‐10‐MALT1 complex, aligning with its anti‐inflammatory mechanism.

To examine the role of BCL‐10 in NF‐κB activity, we compared single‐cell sequencing data between BCL‐10 KO and WT mice. In the BCL‐10^f/f^ CLP group, transcription factors NF‐κB2, RELB, and REL were upregulated, while in BCL‐10^−/−^ CLP mice, their activity was significantly reduced (Figure 9F). In BCL‐10^f/f^ mice, p‐p65 protein expression showed marked dynamic changes. Compared to the Sham group, the CLP‐induced sepsis model group exhibited significant upregulation (*P* < 0.0001, Figure 9G), while emodin intervention effectively suppressed this increase (*P* < 0.001). Notably, in BCL‐10^−/−^ mice, no statistically significant differences in p‐p65 expression were observed among the Sham, CLP, and emodin‐treated groups (*P* = 0.845). Furthermore, the BCL‐10^−/−^ CLP group displayed significantly lower p‐p65 expression levels compared to the BCL‐10^f/f^ CLP group (*P* < 0.0001, Figure S6A, Supporting Information). This regulatory pattern was highly consistent with MPO and NE protein expressions (Figure S6B,C, Supporting Information). In summary, the BCL‐10‐MALT1 complex is a critical regulator of NF‐κB activation and NETs formation. Emodin effectively targets BCL‐10 to inhibit NF‐κB activation, thereby reducing NETs formation, highlighting its therapeutic potential in sepsis.

## 3 Discussion

This study demonstrates that the abnormal activation of inflammation and coagulation is a hallmark of sepsis, with the formation of NETs playing a pivotal role in the interaction between these two processes. Our data show that Emodin significantly inhibits the excessive inflammatory response and abnormal coagulation activation induced by CLP in sepsis, providing substantial protection against multiorgan damage. Moreover, we elucidate the underlying mechanism of Emodin's protective effects. Specifically, Emodin directly binds to BCL‐10 protein, modulating the MALT1/BCL‐10 complex, which leads to the suppression of NF‐κB transcription factor activation and phosphorylation. This results in a reduction in NETs formation and promotes tissue repair in affected organs. Importantly, we also found that the selective depletion of BCL‐10 in neutrophils significantly mitigates sepsis‐induced organ damage and NETs formation. In conclusion, these findings suggest that Emodin, through targeting BCL‐10 and inhibiting NF‐κB phosphorylation‐mediated NETs formation, regulates the balance between inflammation and coagulation, ultimately exerting organ‐protective effects. Our study provides crucial experimental evidence supporting Emodin as a promising therapeutic agent for the treatment of sepsis, expanding its potential applications in clinical settings.

Inflammatory responses and abnormal coagulation activation are hallmark pathological features of sepsis. The CLP model in rodents has been widely accepted as the “gold standard” for studying sepsis. By adjusting the number of perforations, needle gauge, and the length of the ligated cecum, models with varying levels of inflammatory response can be established.^[^ ^23^ ^]^ In this study, we constructed a severe sepsis model to observe the pathological mechanisms of excessive inflammatory responses in sepsis. Excessive inflammation can lead to a “cytokine storm,” which is essentially a self‐amplifying syndrome of proinflammatory cytokine release. Patients typically present with fever, refractory shock, acidosis, and hypermetabolic states, which are major causes of sepsis morbidity and mortality. The pathological mechanism of sepsis‐induced “cytokine storm” may involve persistent activation of innate immunity and suppression of adaptive immunity. TLR4 signaling is considered a key pathway in the pathophysiology of sepsis, mediating innate immune activation. On the one hand, upon pathogen invasion, TLRs are responsible for recognizing PAMPs, which include bacterial components such as LPS, lipopeptides, and lipoteichoic acids. TLRs bind to adaptor proteins with Toll‐interleukin receptor domains, triggering innate immune cells such as monocytes and neutrophils to release large amounts of inflammatory cytokines, type I interferons, and chemokines, thus driving the inflammatory response.^[^ ^24^ ^]^ On the other hand, TLRs also recognize DAMPs, which include proteins, lipids, nucleic acids, and metabolites that amplify the inflammatory response.^[^ ^25^ ^]^ DAMPs activate innate immune cells, such as neutrophils, macrophages, and dendritic cells, as well as nonimmune cells like epithelial and endothelial cells. This activation leads to the release of cytokines and chemokines, further recruiting immune cells and activating adaptive immune responses.^[^ ^26^ ^]^ Our study further corroborates these mechanisms. Single‐cell sequencing results showed a significant increase in neutrophils in the CLP model group compared to the Sham group, suggesting that neutrophil recruitment is a major feature of the early pathological process in sepsis. In addition, high‐throughput detection revealed a significant upregulation of several proinflammatory cytokines and chemokines, such as IFN‐γ, TNF‐α, CXCL1, and CXCL10, 24 h post‐CLP. Moreover, sepsis, as a multiorgan dysfunction syndrome, involves widespread adaptive immune suppression. Studies have shown that patients who succumb to sepsis exhibit marked immunosuppression, characterized by significant reductions in immune cells, such as CD4^+^ and CD8^+^ T cells, in the spleen or lungs. Our results aligning with these findings, as single‐cell sequencing demonstrated a significant decrease in CD8^+^ effector T cells in the CLP model group compared to the Sham group. These results indicate that during the early stages of sepsis, simultaneous innate immune overactivation and adaptive immune suppression contribute to disease progression.

DIC is a common complication in sepsis, occurring in ≈30–51% of patients and strongly associated with increased mortality.^[^ ^27^ ^]^ This syndrome is characterized by widespread activation of coagulation pathways, highlighting that abnormal coagulation cascade activation, or immunocoagulation, is a significant feature in the pathology of sepsis. Our findings further validate this, as high‐throughput screening revealed significant upregulation of several coagulation‐related factors such as Thrombomodulin, Tissue Factor, and GPVI in mice 48 h post‐CLP surgery. The interaction between inflammation and coagulation is a crucial component of innate immunity, serving as a first line of defense against infection. However, excessive inflammatory responses during sepsis often trigger abnormal activation of coagulation and immune thrombosis.^[^ ^28^, ^29^ ^]^ PAMPs and DAMPs can induce tissue factor expression on monocytes, increasing procoagulant activity and promoting platelet aggregation. Platelet aggregation and activation are early indicators of sepsis, and studies have shown that decreased platelet count in DIC patients correlates with poor sepsis outcomes. Additionally, activated platelets release significant amounts of HMGB1, a key mediator of thrombosis.^[^ ^9^, ^30^ ^]^ Elevated serum levels of HMGB1 in sepsis patients are strongly linked to poor prognosis.^[^ ^31^ ^]^ HMGB1, as both a nuclear factor and a secreted protein, regulates innate immune responses within and outside cells. Its interactions with TLR2, TLR4, and RAGE drive inflammation, contributing to the cytokine storm in sepsis and promoting multiorgan damage. Moreover, HMGB1 plays a crucial role in the abnormal activation of the coagulation system, forming a vicious cycle that exacerbates the progression of sepsis.^[^ ^32^ ^]^ In our study, single‐cell sequencing showed significant upregulation of HMGB1 in the CLP group compared to the Sham group. KEGG enrichment analysis revealed notable activation of TLRs , NLRs, RLRs, and platelet activation pathways, indicating that innate immune‐driven inflammation was rapidly initiated. Histopathological analysis also confirmed that CLP‐induced pathological damage to multiple organs. Remarkably, intervention with Emodin significantly reduced the levels of inflammatory cytokines and mitigated abnormal coagulation responses, suggesting that Emodin has the potential to modulate the inflammation–coagulation interplay in sepsis and protect against multiorgan damage.

NETs serve as a critical mediator of the crosstalk between inflammation and coagulation, playing a central role in sepsis pathology, and are also a key target in the intervention of sepsis by Emodin. NETs are web‐like structures composed of DNA, granule proteins, and antimicrobial enzymes released by activated neutrophil. These structures create a physical barrier that traps pathogens, preventing their dissemination. However, persistent inflammation promotes excessive NETs formation, leading to direct damage to host tissues and further amplification of the inflammatory response. NETs have been observed in abundance in sepsis patients. Research indicates that NETs components, such as CitH3, are significantly elevated in the plasma of patients with sepsis‐associated acute pancreatitis and correlate with disease severity.^[^ ^33^ ^]^ Additionally, elevated levels of cfDNA and CitH3 are observed in patients with burn‐induced sepsis, accompanied by a reduction in neutrophil phagocytic capacity.^[^ ^34^ ^]^ cfDNA is considered a biomarker for sepsis severity, with higher levels observed in patients with septic shock compared to those with less severe forms of sepsis.^[^ ^35^, ^36^ ^]^ Furthermore, studies have shown that incubating neutrophils with the plasma or serum of sepsis patients can directly induce NETs formation.^[^ ^37^ ^]^ NETs not only exacerbate tissue damage but also promote immunothrombosis, bridging inflammation and coagulation. NETs can activate ECs through IL‐1α and cathepsin G, leading to the production of tissue factor and ECs activation.^[^ ^38^ ^]^ Activated ECs amplify innate immune responses by producing cytokines, inducing transcription of proinflammatory and oxidative genes, and creating a chemotactic gradient for immune cell recruitment. They also increase P‐selectin secretion, facilitating neutrophil transmigration across the endothelium.^[^ ^16^ ^]^ Disruption of endothelial structure and function is a key factor in immunothrombosis. Furthermore, NETs promote platelet adhesion, activation, and aggregation, providing a scaffold for thrombus formation.^[^ ^12^ ^]^ In this study, we observed widespread NETs formation in CLP‐induced septic mice, and Emodin demonstrated a significant ability to inhibit NETs formation both in vitro and in vivo. Therefore, we propose that Emodin may restore the balance between inflammation and coagulation by targeting NETs, a crucial mediator of this pathological crosstalk.

More importantly, for the first time, our study demonstrated the binding proteins of Emodin through proteomic microarray analysis, identifying BCL‐10 as a potential target. SPR analysis confirmed that Emodin binds to recombinant BCL‐10 protein, a finding further validated through pull‐down assays. However, no previous studies have explored the role of BCL‐10 in the formation of NETs. A literature review revealed that BCL‐10 plays a critical role in immune system activation, primarily by promoting the activation of the nuclear transcription factor NF‐κB. Mice deficient in BCL‐10 exhibit immune deficiencies due to impaired NF‐κB activation, which consequently hampers immune cell activation. Moreover, it is known that the CARD forms a complex with mucosa‐associated lymphoid tissue lymphoma translocation protein 1 (MALT1) and BCL‐10, termed the CBM complex, which activates the NF‐κB pathway and regulates genes related to immune response, inflammation, and cell survival.^[^ ^11^ ^]^ NF‐κB is regarded as a central mediator of inflammation and has been shown to induce NETs formation during sepsis, facilitating the interaction between inflammation and coagulation, which drives immunothrombosis.^[^ ^39^ ^]^ Activation of CBM complex signaling is a highly regulated process, involving multiple post‐translational modifications, and it operates through different pathways depending on the immune cell type. In myeloid cells, the BCL‐10/MALT1 complex controls innate immunity and induces inflammatory cytokine production in response to PAMPs and DAMPs, thereby promoting neutrophil activation.^[^ ^25^ ^]^ Previous studies on the CBM complex have primarily focused on T cells and tumor‐related diseases.^[^ ^40^, ^41^, ^42^ ^]^ In this study, we provide novel insights into the role of the BCL‐10/MALT1 complex in the pathogenesis of sepsis. Western blot and single‐cell sequencing revealed a significant upregulation of BCL‐10 expression in CLP‐induced septic mice, accompanied by increased NF‐κB phosphorylation. Immunofluorescence confirmed the colocalization of the BCL‐10‐MALT1 complex, indicating that elevated BCL‐10 protein activates NF‐κB signaling through the BCL‐10‐MALT1 complex, promoting NETs formation during sepsis. Notably, Emodin targets and suppresses BCL‐10 expression, inhibits NF‐κB phosphorylation, and reduces NETs formation, thereby providing multiorgan protective effects. Remarkably, BCL‐10 knockout in CLP‐induced septic mice led to a marked reduction in NF‐κB signaling, decreased NETs formation, and alleviated organ damage, further supporting that the protective effects of Emodin in sepsis are mediated by modulating the function of the BCL‐10‐MALT1 complex to regulate NF‐κB activation and NETs formation.

While this study systematically elucidates Emodin's therapeutic mechanism of regulating NETosis via BCL‐10 targeting, several limitations warrant further investigation. First, although the current research focuses on efficacy and mechanisms during the therapeutic window period (7 days), long‐term toxicity assessments remain outstanding and will be prioritized in future studies. Furthermore, evidence indicates that glucuronidation metabolism critically contributes to Emodin's low bioavailability.^[^ ^43^ ^]^ In this study, intraperitoneal administration resulted in rapid peak plasma concentration (*T*_max_ = 1 h) and a short elimination half‐life (*t*_1/2z_ = 2.544 h), underscoring the need to develop nanocarriers or prodrug strategies to prolong systemic retention and enhance clinical applicability. Finally, while this work centers on the BCL‐10/NETosis regulatory axis, sepsis pathogenesis inherently involves dynamic interplay among inflammatory activation, coagulation disorders, immune suppression, and metabolic dysregulation. The spatiotemporal crosstalk between metabolic and immune perturbations in sepsis remains incompletely resolved. Future investigations will integrate single‐cell metabolomics with spatial transcriptomics to dynamically map causal–temporal relationships between NETosis and metabolic perturbations, thereby advancing a theoretical framework for multitarget combinatorial therapies.

## 4 Conclusions

In conclusion, our study demonstrates that Emodin exerts significant multiorgan protective effects in sepsis by targeting BCL‐10, thereby modulating the function of the BCL‐10‐MALT1 complex, inhibiting the activation of NF‐κB, and reducing the formation of NETs. These findings provide key evidence that Emodin mitigates sepsis through BCL‐10 inhibition. However, further research is required to evaluate its clinical application. Notably, BCL‐10 may not be the sole target of Emodin, as the compound seems to interact with multiple target proteins. Given this, we carefully interpreted the data and did not overstate BCL‐10 as a specific target. This study not only opens new avenues for investigating Emodin's therapeutic potential but also highlights the importance of exploring its multiple‐target mechanisms for broader applications in sepsis treatment.

## 5 Experimental Section

### Regents

Emodin (99.20% HPLC) (HY‐14393) and DXMS sodium phosphate (HY‐B1829A) were purchased from MedchemExpress (New Jersey, USA). The QAM‐CUST (custom quantitative chip) assay kit was acquired from RayBiotech (Atlanta, USA). HuProt 20K human proteome microarrays were sourced from Huaying Biotechnology (Shanghai, China). The ProcartaPlex Panel multifactor and chemokine detection kit (EPXR360‐26087‐901) and Quant‐iT PicoGreen dsDNA reagent and kits (P11496) were purchased from Thermo Scientific (MA, USA). Anti‐NE antibody (89241), MPO rabbit mAb (14569), CitH3 rabbit mAb (97272), BCL‐10 rabbit mAb (4237), p‐IKKα/β rabbit mAb (2697), p‐IκBα rabbit mAb (2859), p‐NF‐κB p65 rabbit mAb (8242), and β‐actin (4970) were all obtained from Cell Signaling Technology (MA, USA). Anti‐MALT1 rabbit mAb (ab283573) was sourced from Abcam (Shanghai, China). Alexa Fluor 647‐labeled goat anti‐rabbit IgG (H + L) (A0468) and the ROS detection kit (S0033S) were purchased from Beyotime Biotechnology (Shanghai, China). The CCK‐8 (CK04) for cell proliferation and cytotoxicity assays was purchased from Dojindo Laboratories (Japan). Wright–Giemsa staining solution (G1020) was acquired from Solarbio (Beijing, China). Sytox Green staining solution (KFS147) was purchased from BioLegend (Beijing, China). Human NE ELISA kit (MM‐0645H2) and Human CitH3 ELISA kit (MM‐13757H2) were obtained from Jiangsu Meimian Industrial Co., Ltd. (Jiangsu, China).

### Cell Culture

TheHL‐60 cell was obtained from the Cell Bank of the Chinese Academy of Sciences. HL‐60 cells were cultured in RPMI 1640 medium at 37 °C in a humidified atmosphere with 5% CO_2_. The culture medium was supplemented with 20% fetal bovine serum, 100 U mL^−1^ penicillin, and 100 U mL^−1^ streptomycin to ensure optimal cell growth and sterility

### Animals

BCL‐10‐floxed mice were generated by GemPharmatech Co., Ltd. (Nanjing, China). Both BCL‐10^f/f^ and mRP8‐Cre mice were in a C57BL/6J background. BCL‐10^flox/flox^ mice were bred with mRP8‐Cre mice for at least two generations, producing BCL‐10^flox/flox^ mRP8‐Cre mice in which BCL‐10 was conditionally knocked out in neutrophils. All healthy, specific pathogen‐free C57BL/6J experimental mice (8–10 weeks, 20–23 g) were obtained from Beijing Huafukang Biotechnology Co., Ltd. The mice were housed under a 12 h light/dark cycle with free access to food and water. Before the experiments, the mice were acclimated to the environment at a temperature of 18–22 °C for one week. The animal study protocol was approved by the Animal Care and Use Committee of the Beijing Institute of Traditional Chinese Medicine (BJTCM‐M‐2022‐10‐01).

### Sepsis Remodeling in Mice

After a one‐week acclimatization period, mice were fasted for 12 h before surgery, with free access to water, and a sepsis model was established using CLP. The procedure was as follows: mice were anesthetized with an intraperitoneal injection of 1% sodium pentobarbital (10 mL kg^−1^) and placed in a supine position. After disinfecting the abdominal area, a left lower quadrant incision was made to expose the cecum, which was then ligated at the proximal one‐quarter point between the base and distal end with silk sutures. A 21G needle was used to puncture the cecum twice, allowing a small amount of fecal material to extrude, after which the cecum was repositioned, and the abdominal wall was closed in layers. Immediately postsurgery, 1 mL of sterile saline was administered for fluid resuscitation, and mice were allowed to recover naturally. The sham‐operated group underwent the same procedure but without cecal ligation or puncture. No antibiotics were administered to any mice, and mortality was monitored for 7 days postoperation.

### Model I: Sepsis Model Induced by CLP and Simultaneous Treatment with Emodin in Wild‐Type Mice

Eight‐week‐old C57BL/6J mice were randomly assigned to four groups: I) Sham‐operated group, treated with 0.1% DMSO as vehicle control (Sham, *n* = 20); II) CLP model group, also treated with 0.1% DMSO vehicle control (CLP, *n* = 20); III) DXMS‐treated group, receiving intraperitoneal injections of 2 mg kg^−1^ DXMS twice daily at 200 µL per dose (CLP + DXMS, *n* = 20); and IV) Emodin‐treated group, receiving intraperitoneal injections of 20 mg kg^−1^ Emodin twice daily at 200 µL per dose (CLP + Emodin, *n* = 20). The initial dose of Emodin, DXMS, or vehicle control was administered once the mice fully recovered from surgery, with treatments continuing for 7 days.

### Model II: Sepsis Model Induced by CLP and Simultaneous Treatment with Emodin in BCL‐10^f/f^ and BCL‐10^−/−^ Mice

Eight‐week‐old BCL‐10^f/f^ and BCL‐10^−/−^ mice were randomly assigned into three groups each, resulting in six total groups: I) BCL‐10^f/f^ sham‐operated group, treated with 0.1% DMSO vehicle control (BCL‐10^f/f^ Sham, *n* = 20); II) BCL‐10^f/f^ CLP model group, treated with 0.1% DMSO vehicle control (BCL‐10^f/f^ CLP, *n* = 20); III) BCL‐10^f/f^ Emodin‐treated group, receiving 20 mg kg^−1^ Emodin via intraperitoneal injection twice daily at 200 µL per injection (BCL‐10^f/f^ CLP + Emodin, *n* = 20); IV) BCL‐10^−/−^ sham‐operated group, treated with 0.1% DMSO vehicle control (BCL‐10^−/−^ Sham, *n* = 20); V) BCL‐10^−/−^ CLP model group, treated with 0.1% DMSO vehicle control (BCL‐10^−/−^ CLP, *n* = 20); and VI) BCL‐10^−/−^ Emodin‐treated group, receiving 20 mg kg^−1^ Emodin via intraperitoneal injection twice daily at 200 µL per injection (BCL‐10^−/−^ CLP + Emodin, *n* = 20). The initial administration of Emodin or vehicle control was provided postsurgery recovery, with treatments maintained for 7 days.

### High‐Throughput Detection of Multiple Inflammatory Cytokines, Chemokines, and Coagulation‐Related Factors

To analyze inflammatory and chemokine factors in mouse serum, the ProcartaPlex multiplex assay kit was utilized with the Luminex 2000 system. Key steps included serum collection, standard dilution preparation per protocol, and microsphere washing. Samples (25 µL each) were loaded, followed by plate washing and addition of detection antibody mixtures and streptavidin–phycoerythrin. Finally, 120 µL of reading buffer was added, and detection proceeded on the Luminex 2000. Target analytes included chemokines like ENA‐78 (CXCL5), Eotaxin (CCL11), GRO‐α (CXCL1), IP‐10 (CXCL10), MCP‐1 (CCL2), and cytokines such as G‐CSF (CSF‐3), GM‐CSF, IFN‐α, IFN‐γ, IL‐1α, IL‐6, IL‐10, and TNF‐α.

For coagulation‐related cytokine detection in mouse plasma, the QAM‐CUST custom chip was employed. Plasma was collected, and after preparing standard dilutions, samples (100 µL each) were loaded and incubated overnight at 4 °C. Postincubation, slides were washed with a chip washing machine, followed by detection antibody mixtures and Cy3‐labeled streptavidin addition. After a 1 h room temperature incubation and another wash, signals were detected using the InnoScan 300 laser scanner at 532 nm wavelength and 10 µm resolution. Analyzed coagulation‐related markers included Thrombomodulin, TF, CD34, P‐selectin, Gas6, GPVI, S100A9, CD40L, and TFPI‐2, among others.

### Human Proteome Microarray Assays

The HuProt 20K Human Proteome Microarray was utilized to identify proteins interacting with Emodin. The chip was initially pretreated with a blocking buffer, then incubated with either 50 µm biotinylated Emodin or free biotin. Following washes, Cy5‐labeled streptavidin was applied, and scanning was conducted using the GenePix 4000B scanner (Axon Instruments, Sunnyvale, CA) to acquire raw data. The signal‐to‐noise ratio (SNR) for each protein was calculated as the ratio of the median foreground to background signal, with the average SNR determined from duplicate spots. SNR values for microarrays treated with biotinylated Emodin and without were designated as SNR (+) and SNR (−), respectively. The “Ratio,” calculated as SNR (+)/SNR (−), provided a measure of signal intensity. Candidate proteins were selected based on a threshold Ratio > 3.0, and data analysis was performed using GenePix Pro v6.0 software.

### Surface Plasmon Resonance Analysis

Using the Biacore T200 protein interaction analysis system (GE Healthcare) with NTA sensor chips (BR100407), the binding affinity between Emodin and recombinant human BCL‐10 (rhBCL‐10) was assessed. The rhBCL‐10 protein was dissolved in 10 mm acetate buffer (pH 5.0) and immobilized on the sensor chip surface using an amine coupling kit. Emodin solutions were prepared at nine concentrations (0, 781.25 nm, 1.5625, 3.125, 6.25, 12.5, 25, 50, and 100 µm) in PBS with 5% DMSO. Samples were analyzed under a flow rate of 30 µL min^−1^ during a 180 s association phase, followed by a 250 s dissociation phase, with a constant temperature of 25 °C. Data were processed using Biacore T200 software, applying global fitting to the kinetic data across Emodin concentrations. Key binding kinetics parameters—association rate constant, dissociation rate constant, and equilibrium dissociation constant—were determined based on a 1:1 Langmuir binding model.

### Emodin‐BCL‐10 Binding Assays

The binding affinity between Emodin and BCL‐10 was evaluated using a protein interaction pull‐down assay with the Pierce Biotinylated Protein Interaction Pull‐Down Kit (Thermo Fisher). To begin, 100 µL of 20 mm biotinylated Emodin was incubated with 50 µL of streptavidin–agarose beads at 4 °C for 30 min, with biotin alone serving as the control. Next, liver tissue lysates from mice were added to the streptavidin–agarose beads preincubated with biotinylated Emodin, and the mixture was gently agitated at 4 °C for 24 h. After incubation, samples were centrifuged and washed three times to remove nonspecific binding. Elution buffer was then added to each spin column, and the eluted products were boiled with 5× loading buffer. Samples were subsequently loaded onto a 10% polyacrylamide gel for WB analysis. Total lysates were used as input controls to confirm protein integrity and validate the reliability of the results.

### Computational Docking and Molecular Simulation

The crystal structure of the BCL‐10‐DNA complex (PDB: 6BEZ) was retrieved from the Protein Data Bank (PDB). Input files for docking, including ligand and receptor structures, were prepared with the Graphical User Interface program AutoDock Tools 1.5.6 (The Scripps Research Institute, USA). Molecular docking was then performed using AutoDock Vina 1.0.2. Predicted docking pockets identified by PyMOL software were subsequently used as the basis for molecular dynamics simulations, conducted with the AMBER14 software package. Key residues involved in protein–ligand interactions were determined through per‐residue decomposition energy analysis.

### Plasmids and Transfection

Streptococcus‐tagged BCL‐10 and its mutants were constructed using SnapGene and subsequently synthesized by YouBio. The PCR‐amplified fragments were inserted into the pCDH‐EF1‐MCS‐T2A‐Puro vector (YouBio) to create Streptococcus‐tagged BCL‐10 and its mutants, with Streptococcus labeling at the N‐terminus. All plasmids were confirmed by sequencing and immunoblotting. These plasmids were then transfected into human embryonic kidney 293T (HEK293T) cells using StarFect transfection reagents (GenStar, C101) according to the manufacturer's instructions.

### Coupling with Epoxy‐Activated Agarose Gel 6B

The coupling of BCL‐10 and rhamnol to epoxy‐activated Sepharose 6B beads (E6754, Sigma‐Aldrich, Shanghai) was performed following the manufacturer's instructions. Initially, 0.6 g of epoxy‐activated Sepharose 6B beads was hydrated in distilled water and subjected to three consecutive washes using the coupling buffer (0.1 m Na_2_CO_3_, pH 9.0). Subsequently, the beads were incubated with emodin (10 mg mL^−1^ in a mixture of 400 µL DMSO and 800 µL coupling buffer) under gentle rotation at 37 °C for an overnight period. After the incubation, the beads were extensively washed to remove any unbound emodin. The resulting emodin‐conjugated Sepharose 6B beads were then collected for further use.

### Co‐Immunoprecipitation and Competitive Inhibition

HEK293T cells were plated in 100 mm culture dishes at a density of 6.0 × 10^6^ cells per dish. Upon achieving full adherence to the dish surface, the cells were transfected with a Flag‐tagged expression plasmid for 17 h at 37 °C. Following transfection, the cells were lysed using a cell lysis buffer composed of 50 mm Tris‐HCl, 1% Triton X‐100, 0.25% sodium deoxycholate, 150 mm NaCl, 0.1% NP‐40, and 2 mm EDTA, pH 7.4. The lysates were collected and subjected to centrifugation at 12 000 × *g* for 15 min at 4 °C. The clarified supernatant was then incubated with emodin‐conjugated Sepharose 6B agarose beads for 16 h at 4 °C with gentle rotation. After incubation, the beads were washed 15 times with the same lysis buffer to remove non‐specific binding. The emodin‐conjugated proteins were subsequently eluted using SDS loading buffer and resolved by sodium dodecyl sulfate‐polyacrylamide gel. The resolved proteins were then transferred to polyvinylidene fluoride or nitrocellulose membranes for detection by immunoblotting. For competitive inhibition experiments, 100 µm free emodin was added to the lysate immediately after mixing with the emodin‐conjugated Sepharose 6B beads. The subsequent steps were performed as described above for the co‐immunoprecipitation experiments.

### Cellular Thermal Shift Assay

1 × 10^7^ PMA‐treated HL‐60 cells (48 h differentiation) were harvested, trypsinized (0.25% trypsin), and washed with PBS. The cell pellet was resuspended in 600 µL of PBS supplemented with 1% protease inhibitor cocktail, then split equally into two tubes. After three freeze–thaw cycles in liquid nitrogen, samples were centrifuged at 14 000 × *g* for 10 min at 4 °C to clarify the supernatants. One aliquot was incubated with 100 µm emodin for 1 h at 37 °C, while the other served as a vehicle control. Both samples were subdivided into seven equal portions and subjected to heat treatment across a temperature gradient (37 °C, 42 °C, 47 °C, 52 °C, 57 °C, 62 °C, and 67 °C) for 3 min each. After heat exposure, samples were centrifuged at 16 000 × *g* for 20 min at 4 °C to pellet aggregated proteins. The supernatants were collected, mixed with 5× SDS loading buffer, and analyzed by WB to quantify BCL‐10 levels at each temperature. This approach enabled us to assess emodin–BCL‐10 binding by monitoring ligand‐induced changes in protein thermal stability.

### Single‐Cell Transcriptome Sequencing

Twenty‐four hours post‐CLP modeling, fresh blood was collected from the Sham, CLP, and Emodin‐treated groups of BCL‐10^f/f^ and BCL‐10^−/−^ mice. Single‐cell suspensions were prepared using the 10× Genomics Chromium Single Cell 3′ Solution. Cell viability, ensured at ≥90%, was confirmed through cell counting prior to RNA‐seq library preparation, following the 10× Genomics standard protocol. High‐throughput sequencing was conducted using the Illumina NovaSeq 6000 system.

For data processing, the Seurat R package was used to filter, normalize, reduce dimensionality, and perform unsupervised clustering of the single‐cell transcriptomic data. Cells expressing more than 8000 genes, fewer than 200 genes, or showing mitochondrial gene expression exceeding 25% were excluded. Dimensionality reduction was performed with the RunPCA function, and primary cell clusters were identified with the FindClusters function. Cluster visualization was achieved using UMAP. Differential gene expression was analyzed using the Wilcoxon rank‐sum test in Seurat, identifying differentially expressed genes across cell subpopulations. These genes were further analyzed with gene ontology, KEGG, Reactome pathway database and disease ontology enrichment analyses to investigate their biological functions and associated pathways.

### Hematoxylin and Eosin Staining

Lung, liver, kidney, and spleen tissues from mice were fixed in 4% paraformaldehyde (PFA) and embedded in paraffin. Subsequently, 5 µm tissue sections were prepared, deparaffinized, and rehydrated on glass slides. H&E staining was performed to evaluate pathological changes in each organ. The stained sections were meticulously examined under a light microscope to identify microscopic pathological features across the organs.

### Tissue and Cell Immunofluorescence Analysis

To assess NETs formation both in vitro and in vivo, IF staining was conducted. For dHL‐60 cells, fixation was achieved with 4% PFA, followed by permeabilization with 0.2% Triton X‐100. To reduce nonspecific binding, cells were blocked with diluted goat serum for 1 h at room temperature. The cells were then incubated with an anti‐NE mAb (1:70) for 1 h, followed by an Alexa Fluor 647‐conjugated goat anti‐rabbit secondary antibody (1:500) at room temperature for another hour. After three washes with PBST, Sytox Green at 0.5 µm was added for nuclear staining at room temperature.

For mouse liver tissue, 5 µm paraffin sections were deparaffinized, rehydrated, subjected to antigen retrieval, and washed. The slides were incubated overnight at 4 °C with primary antibodies against MPO (1:50), CitH3 (1:200), MALT1 (1:100), and BCL‐10 (1:100). After washing, sections were incubated with FITC, Cy3, and Cy5.5‐conjugated secondary antibodies at room temperature for 1 h, followed by mounting with an antifade medium containing DAPI. IF staining was observed and imaged using a Zeiss laser confocal fluorescence microscope (ZEISS, LSM 880, Germany).

### Western Blotting Analysis

Liver tissue samples were lysed and quantified using a BCA protein assay, with 50 µg of protein loaded per sample. Proteins were separated on a 4–20% SDS‐PAGE and transferred to PVDF membranes. The membranes were blocked with 5% nonfat milk at room temperature for 1 h to minimize nonspecific binding. Next, the PVDF membranes were incubated overnight with primary antibodies targeting the following proteins: anti‐NE mAb (1:1000), MPO rabbit mAb (1:1000), CitH3 rabbit mAb (1:1000), BCL‐10 rabbit mAb (1:1000), p‐IKKα/β rabbit mAb (1:1000), p‐IκBα rabbit mAb (1:1000), p‐p65 rabbit mAb (1:1000), and β‐actin mAb (1:1000). The membranes were then incubated with horseradish peroxidase‐conjugated secondary antibodies and enhanced chemiluminescence reagents. Imaging was conducted using the Odyssey dual‐color infrared laser imaging system, and densitometric analysis for quantification was performed with ImageJ software.

### Induction of NETs Formation in dHL‐60 Cells by PMA

Logarithmically growing dHL‐60 cells were seeded in a 96‐well plate at a density of 1 × 10^5^ cells per well. Experimental groups included a blank control, a negative control, a solvent control, and PMA treatment groups at concentrations of 10, 20, 50, 80, 100, 200, and 500 ng mL^−1^, with six replicates per group. Cells were stimulated by 1.5, 3, and 5 h, and intracellular ROS fluorescence intensity was measured using the DCFH‐DA probe and a fluorescent microplate reader (excitation: 488 nm; emission: 525 nm). This setup allowed determination of the optimal PMA concentration and duration for inducing NETs formation in dHL‐60 cells.

### Cell Viability

Cell viability was assessed using the CCK‐8 assay. For this assay, logarithmically growing dHL‐60 cells were prepared in fresh serum‐free medium or medium containing different concentrations of drugs to create a cell suspension. Cells were seeded into 96‐well plates at a density of 1 × 10^4^ cells per well, with Emodin concentrations set at eight gradients (0, 1, 2, 5, 10, 20, 50, and 100 µm). Following incubation at 37 °C with 5% CO_2_ and saturated humidity for 1.5, 3, and 5 h, the medium was removed by centrifugation. Next, 10 µL of CCK‐8 solution was added in the dark, and cells were incubated for an additional 1.5 h. Absorbance was measured at 450 nm to assess the effect of Emodin on dHL‐60 cell viability.

The CCK‐8 assay was also used to determine the impact of Emodin on dHL‐60 cell survival under PMA stimulation. Logarithmically growing dHL‐60 cells were seeded into 96‐well plates, with experimental groups comprising a control group, a PMA stimulation group, and Emodin treatment groups (1, 2, 5, 10, 20, 50, and 100 µm). Absorbance was measured according to the same protocol to evaluate cell viability across conditions.

### Statistical Analysis

Statistical analyses were conducted using GraphPad Prism 9.5.2 (CA, USA). Data normality was first assessed with the Shapiro–Wilk test. For normally distributed data, comparisons between two groups were analyzed with a two‐tailed Student's *t*‐test, while comparisons among three or more groups used one‐way ANOVA followed by Tukey's post hoc test. For non‐normally distributed data, nonparametric tests were applied: the Mann–Whitney U test for two‐group comparisons and the Kruskal–Wallis test with Dunn's post hoc analysis for groups of three or more. For multiple measurements from the same mouse, two‐way repeated‐measures ANOVA with a single pooled variance was used, applying Tukey's correction for pairwise comparisons within groups. Statistical significance was set at *P* ≤ 0.05.

## Conflict of Interest

The authors declare no conflict of interest.

## Author Contributions

X.X., Y.Y., and M.Z. contributed equally to this work. W.G. and Q.L. contributed to the literature search and study design. X.X., Y.Y., M.Z., M.Z., T.C., H.Q., Y.B., S.Z., C.Z., Y.S., Y.L., N.W., Y.B., Y.Z., and Z.B. performed the experimental operations. X.X., Y.Y., and M.Z. participated in the drafting of the article. W.G. and Q.L. revised the manuscript.

## Supporting information



Supporting Information

## Data Availability

Research data are not shared.
