# Supplementary material for: Emodin Alleviates Sepsis‐Induced Multiorgan Damage by Inhibiting NETosis through Targeting Neutrophils BCL‐10
Source: Adv Sci (Weinh). 2025 Aug 8;12(41):e17129. doi: 10.1002/advs.202417129 (PMC12591137; doi:10.1002/advs.202417129)
Supplement: Supplementary file 1 — Supporting Information [file ADVS-12-e17129-s001.docx]

**
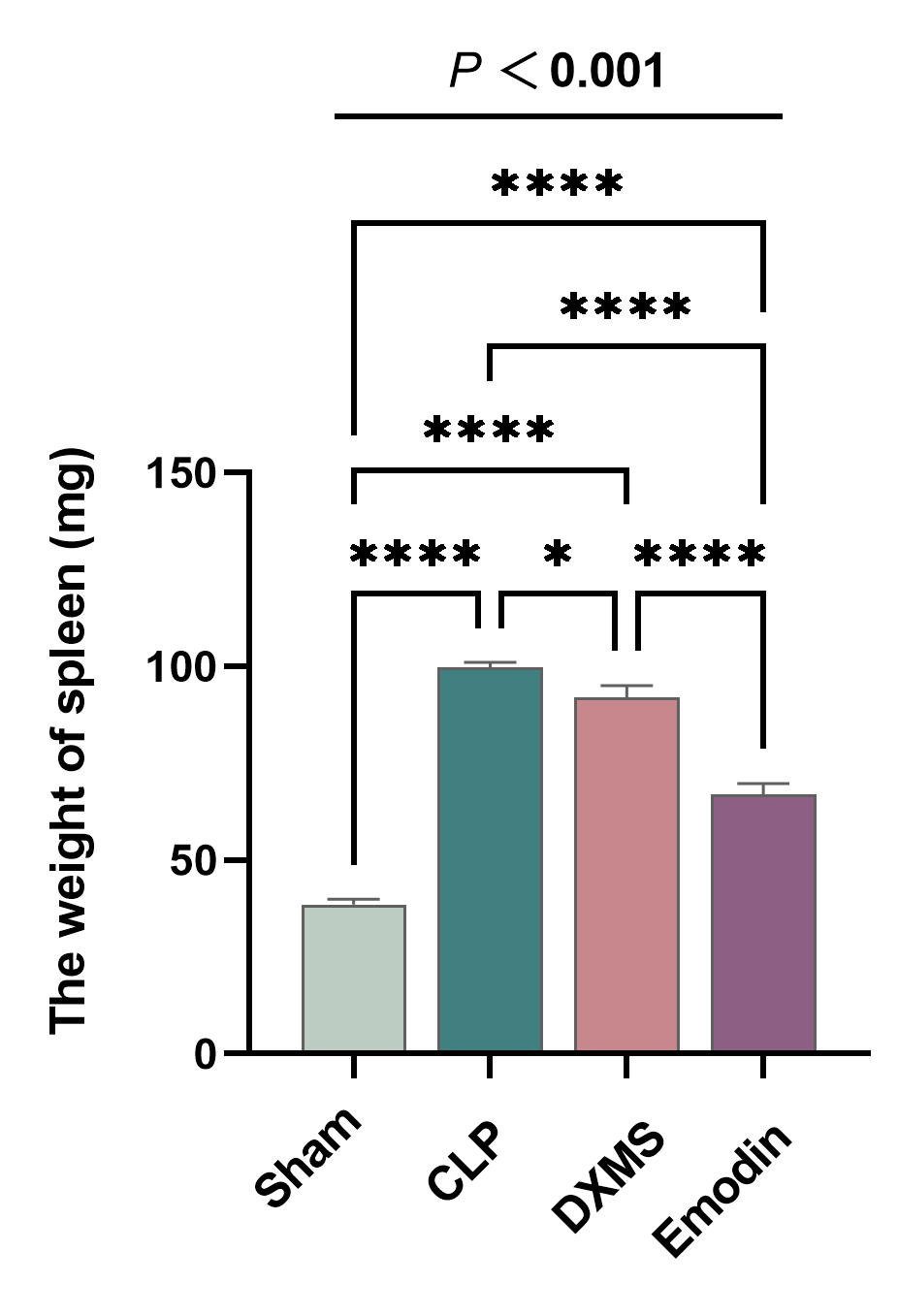
**

**Figure S1.** Bar graph of spleen weight in the four experimental groups of mice. **P* < 0.05, ***P* < 0.01, ****P* < 0.001, ****P* < 0.0001*;* n.s., no significant difference (*P* > 0.05)*.*

**Table S1. The effect of emodin on cfDNA expression levels in septic mice**

| Time | Group | | | Total | F | *P* |
| --- | --- | --- | --- | --- | --- | --- |
|  | Sham | CLP | Emodin |  |  |  |
| 24 h | 34.56 ± 0.87 | 57.84 ± 1.21^****^ | 41.29 ± 1.36^####^ | 44.75 ± 10.39 | 975.88 | ＜0.0001 |
| 48 h | 33.69 ± 0.56 | 37.31 ± 0.54^****^ | 36.85 ± 0.40 | 35.95 ± 1.72 | 26.53 | ＜0.0001 |
| 72 h | 34.13 ± 0.72 | 38.27 ± 0.68^****^ | 35.84 ± 1.50^####^ | 36.08 ± 2.01 | 29.75 | ＜0.0001 |
| Total | 34.13 ± 0.78 | 44.47 ± 9.76 | 37.80 ± 2.61 | 38.82 ± 7.24 | 560.88^a^ | 0.000^a^ |
| F | 1.293 | 918.466 | 50.939 | 478.72^a^ | 236.82^b^ | ＜0.0001^b^ |
| *P* | 0.285 | ＜0.0001 | ＜0.0001 | 0.000^a^ |  |  |

a, the F-statistic and *P*-value for the main effect. b, the F-statistic and P-value for the interaction effect. Compared to the Sham group, * *P* < 0.05, ** *P* < 0.01, *** *P* < 0.001, **** *P* < 0.0001; compared to the CLP group, # *P* < 0.05, ## *P* < 0.01, ### *P* < 0.001, #### *P* < 0.0001. All data in the table have been normalized to a normal distribution from the original data.

**Table S2. The effect of emodin on NE protein expression levels in septic mice**

| Time | Group | | | Total | F | *P* |
| --- | --- | --- | --- | --- | --- | --- |
|  | Sham | CLP | Emodin |  |  |  |
| 24 h | -1.00 ± 0.40 | 0.14 ± 0.22^****^ | -0.67 ± 0.25^##^ | -0.55 ± 0.57 | 17.04 | ＜0.0001 |
| 48 h | -1.11 ± 0.26 | -0.41 ± 0.17^**^ | -0.68 ± 0.18 | -0.76 ± 0.37 | 7.73 | 0.002 |
| 72 h | -2.02 ± 0.40 | -0.50 ± 0.29^****^ | -0.67 ± 0.39 | -0.95 ± 0.73 | 26.53 | ＜0.0001 |
| Total | -1.27 ± 0.52 | -0.27 ± 0.35 | -0.67 ± 0.27 | -0.75 ± 0.57 | 47.03^a^ | 0.000^a^ |
| *F* | 12.551 | 5.666 | 0.003 | 10.649^a^ | 4.71^b^ | 0.004^b^ |
| *P* | ＜ 0.001 | 0.008 | 0.997 | ＜ 0.001^a^ |  |  |

a, the F-statistic and P-value for the main effect. b, the F-statistic and *P*-value for the interaction effect. Compared to the Sham group, * *P* < 0.05, ** *P* < 0.01, *** *P* < 0.001, **** *P* < 0.0001; compared to the CLP group, # *P* < 0.05, ## *P* < 0.01, ### *P* < 0.001, #### *P* < 0.0001. All data in the table have been normalized to a normal distribution from the original data.

**Table S3. The effect of emodin on CitH3 protein expression levels in septic mice**

| Time | Group | | | Total | F | *P* |
| --- | --- | --- | --- | --- | --- | --- |
|  | Sham | CLP | Emodin |  |  |  |
| 24 h | 0.55 ± 0.06 | 0.98 ± 0.08^****^ | 0.89 ± 0.09 | 0.82 ± 0.20 | 29.22 | ＜0.0001 |
| 48 h | 0.77 ± 0.04 | 0.95 ± 0.13^**^ | 0.81 ± 0.09^#^ | 0.85 ± 0.12 | 5.21 | 0.009 |
| 72 h | 0.81 ± 0.12 | 0.89 ± 0.09 | 0.81 ± 0.10^#^ | 0.81 ± 0.12 | 3.811 | 0.03 |
| Total | 0.72 ± 0.14 | 0.94 ± 0.10 | 0.81 ± 0.11 | 0.83 ± 0.15 | 24.451^a^ | ＜0.0001^a^ |
| *F* | 10.989 | 1.173 | 3.334 | 0.793^a^ | 7.50^b^ | ＜0.001^b^ |
| *P* | ＜ 0.001 | 0.319 | 0.045 | 0.459^a^ |  |  |

a, the F-statistic and P-value for the main effect. b, the *F*-statistic and *P*-value for the interaction effect. Compared to the Sham group, * *P* < 0.05, ** *P* < 0.01, *** *P* < 0.001, **** *P* < 0.0001; compared to the CLP group, # *P* < 0.05, ## *P* < 0.01, ### *P* < 0.001, #### *P* < 0.0001. All data in the table have been normalized to a normal distribution from the original data.

**Table S4. Main and interaction effects of emodin on MPO expression levels in septic mice**

| Source of Variation | Mean Square | F | *P* | Partial η^2^ |
| --- | --- | --- | --- | --- |
| Group | 1.411 | 68.724 | ＜ 0.0001 | 0.821 |
| Time | 0.073 | 3.548 | 0.041 | 0.191 |
| Group*Time | 0.041 | 1.980 | 0.123 | 0.209 |

**
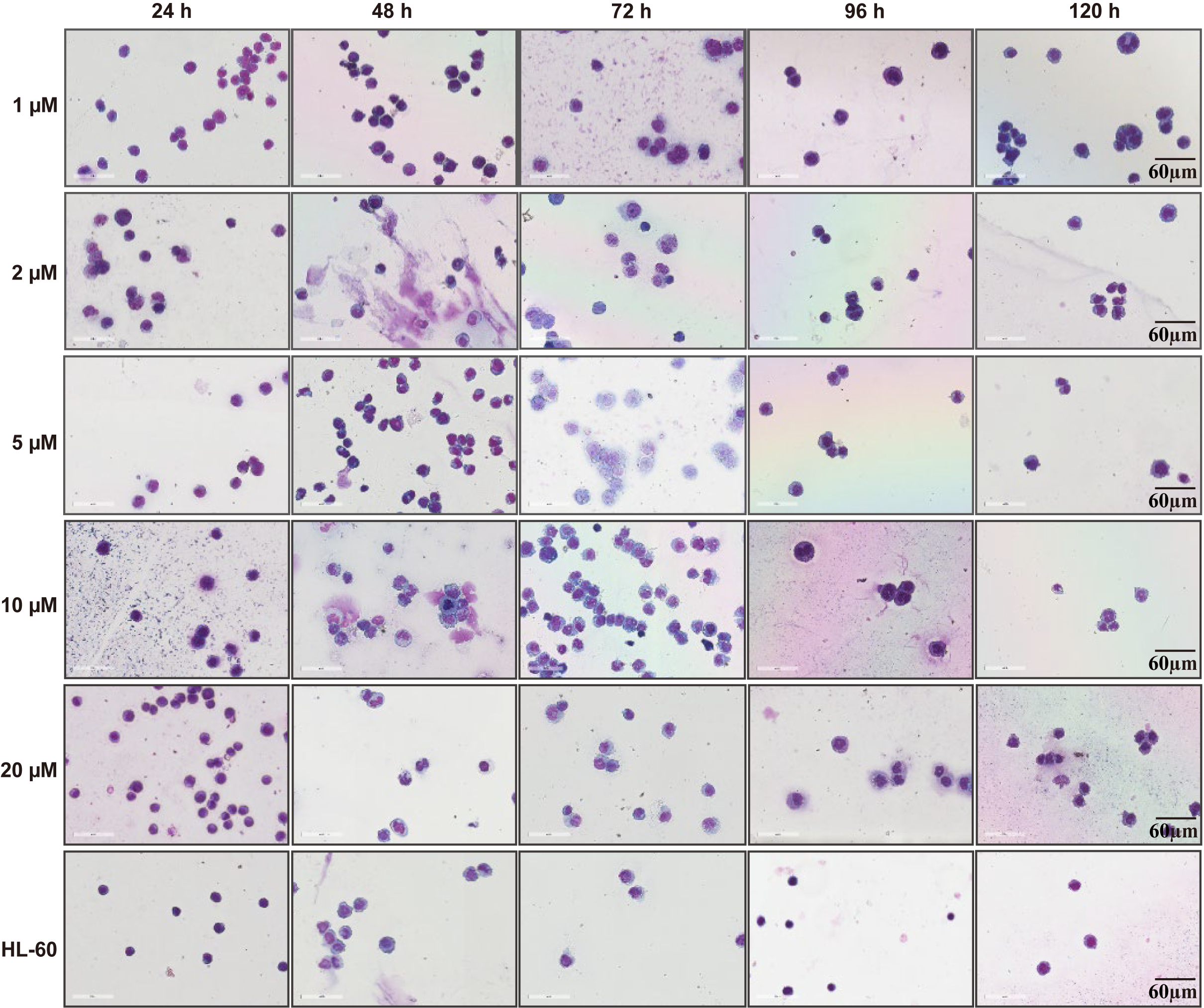
**

**Figure S2. Wright-Giemsa stained to observe the morphology of ATRA-induced differentiation of HL-60 cells into dHL-60 cells.**

**
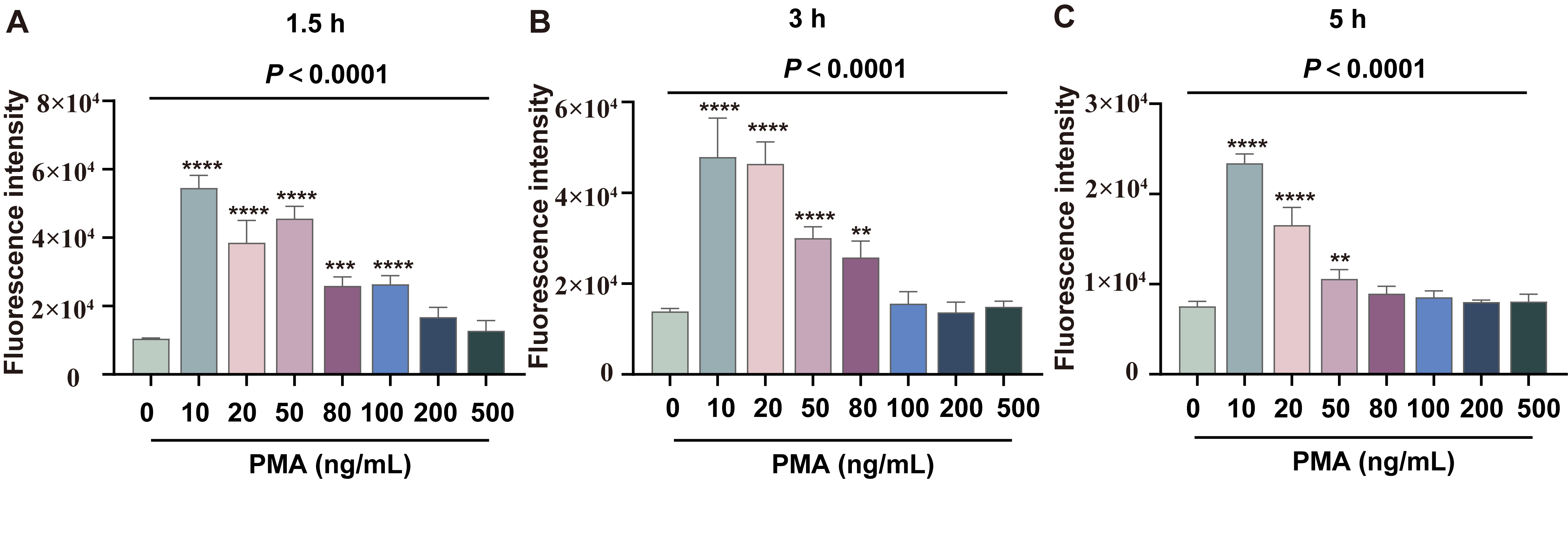
**

**Figure S3. ROS production in dHL-60 cells stimulated with PMA at different time points and concentrations.** A-C. ROS levels in dHL-60 cells stimulated with varying concentrations of PMA (0, 10, 20, 50, 80, 100, 200, and 500 ng/mL) for 1.5 h (A), 3 h (B), and 5 h (C). Compared with the non-PMA stimulation group: **P* < 0.05, ***P* < 0.01, ****P* < 0.001, *****P* < 0.0001.


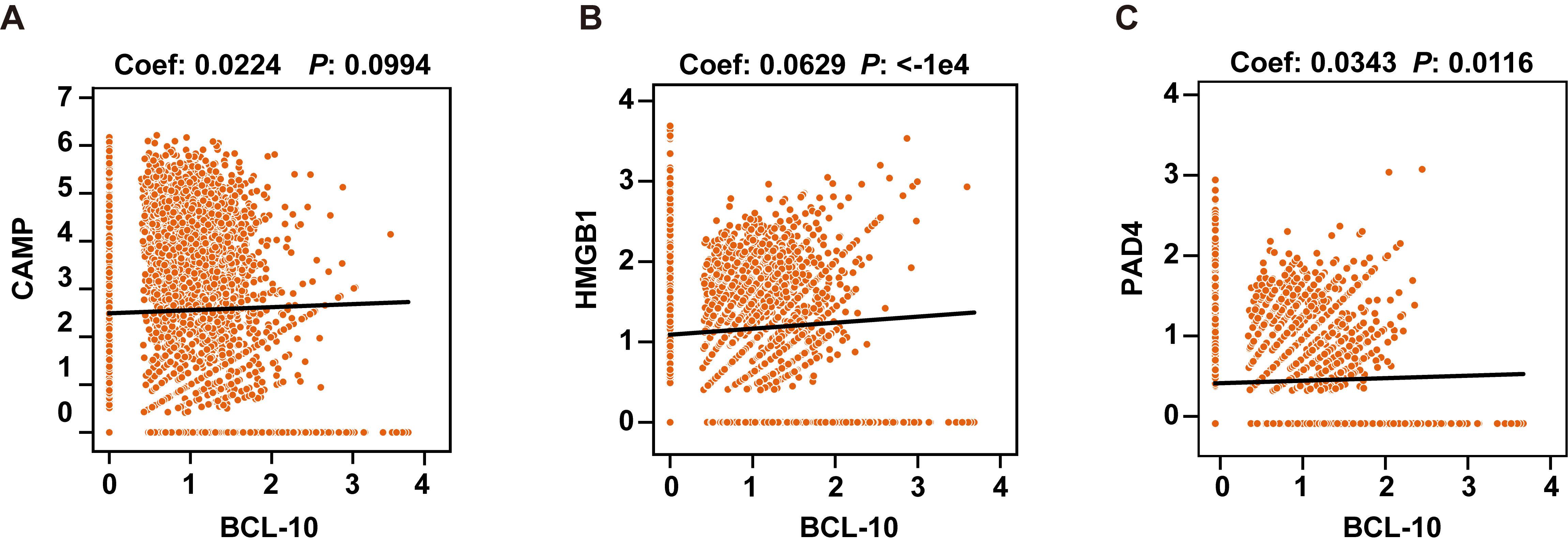


**Figure S4. Positive correlation between BCL-10 and NETs-related genes.** A-C. Pearson correlation analysis between BCL-10 and CAMP (A), HMGB1 (B), and PAD4 (C). *P* < 0.05 was considered statistically significant.

**Table S5. Main and interaction effects of emodin on p-p65 expression levels in septic mice**

| Source of variation | Mean square | F | *P* | Partial η^2^ |
| --- | --- | --- | --- | --- |
| Groups | 0.526 | 19.792 | ＜ 0.0001 | 0.586 |
| Time | 0.013 | 0.505 | 0.609 | 0.035 |
| Groups*Time | 0.033 | 1.252 | 0.312 | 0.152 |

**Table S6. Effect of emodin on NF-κB p65 phosphorylation levels in septic mice.**

| Time/Group | Sham | CLP | Emodin | F | *P* |
| --- | --- | --- | --- | --- | --- |
| 24 h | 0.54 ± 0.07 | 0.86 ± 0.13^**^ | 0.73 ± 0.10 | 6.997 | 0.015 |
| 48 h | 0.56 ± 0.07 | 0.84 ± 0.12^**^ | 0.60 ± 0.07^##^ | 10.605 | 0.003 |
| 72 h | 0.64 ± 0.12 | 0.85 ± 0.13^*^ | 0.55 ± 0.17^##^ | 5.953 | 0.023 |
| Weight mean | 0.34 ± 0.04 | 0.73 ± 0.05^****^ | 0.41 ± 0.05^####^ | - | - |

Compared to the Sham group: * *P* ＜ 0.05, ** *P* ＜ 0.01, *** *P* ＜ 0.001, **** *P* ＜ 0.0001; compared to the CLP group: # *P* ＜ 0.05, ## *P* ＜ 0.01, ### *P* ＜ 0.001, #### *P* ＜ 0.0001.

**Table S7. Main and interaction effects of emodin on** **IKBα phosphorylation levels in septic mice**

| Source of variation | Mean square | F | *P* | Partial η^2^ |
| --- | --- | --- | --- | --- |
| Groups | 0.442 | 27.226 | ＜ 0.0001 | 0.616 |
| Time | 0.001 | 0.041 | 0.960 | 0.002 |
| Group*Time | 0.014 | 0.834 | 0.513 | 0.089 |

**Table S8. Effect of emodin on IKBα phosphorylation levels in septic mice.**

| Time/Group | Sham | CLP | Emodin | F | *P* |
| --- | --- | --- | --- | --- | --- |
| 24 h | 0.54 ± 0.07 | 0.86 ± 0.13^*^ | 0.73 ± 0.10 | 6.997 | 0.015 |
| 48 h | 0.56 ± 0.07 | 0.84 ± 0.12^**^ | 0.60 ± 0.07^##^ | 10.605 | 0.003 |
| 72 h | 0.64 ± 0.12 | 0.85 ± 0.13^**^ | 0.55 ± 0.17^#^ | 5.953 | 0.023 |
| Weight mean | 0.37 ± 0.03 | 0.72 ± 0.04^****^ | 0.47 ± 0.04^####^ | - |  |

Compared to the Sham group: * *P* ＜ 0.05, ** *P* ＜ 0.01, *** *P* ＜ 0.001, **** *P* ＜ 0.0001; compared to the CLP group: # *P* ＜ 0.05, ## *P* ＜ 0.01, ### *P* ＜ 0.001, #### *P* ＜ 0.0001.

**Table S9. The effect of emodin on IKKα/β protein phosphorylation levels in septic mice**

| Time | Group | | | Total | F | *P* |
| --- | --- | --- | --- | --- | --- | --- |
|  | Sham | CLP | Emodin |  |  |  |
| 24 h | 0.19 ± 0.11 | 0.52 ± 0.10^****^ | 0.40 ± 0.13 | 0.37 ± 0.18 | 13.97 | ＜0.0001 |
| 48 h | 0.21 ± 0.02 | 0.62 ± 0.01^****^ | 0.29 ± 0.12^####^ | 0.34 ± 0.19 | 21.73 | ＜0.0001 |
| 72 h | 0.19 ± 0.04 | 0.63 ± 0.11^****^ | 0.21 ± 0.09^####^ | 0.31 ± 0.21 | 27.73 | ＜0.0001 |
| Total | 0.20 ± 0.06 | 0.59 ± 0.10 | 0.29 ± 0.13 | 38.82 ± 7.24 | 59.70^a^ | ＜0.0001^a^ |
| *F* | 10.989 | 1.173 | 3.334 | 0.793^a^ | 2.78^b^ | 0.048^b^ |
| *P* | ＜ 0.001 | 0.319 | 0.045 | 0.459^a^ |  |  |

a, the F-statistic and P-value for the main effect. b, the F-statistic and *P*-value for the interaction effect. Compared to the Sham group, * *P* < 0.05, ** *P* < 0.01, *** *P* < 0.001, **** *P* < 0.0001; compared to the CLP group, # *P* < 0.05, ## *P* < 0.01, ### *P* < 0.001, #### *P* < 0.0001.

**Table S10. The effect of emodin on BCL-10 protein levels in septic mice**

| Time | Group | | | Total | F | *P* |
| --- | --- | --- | --- | --- | --- | --- |
|  | Sham | CLP | Emodin |  |  |  |
| 24 h | 0.36 ± 0.11 | 0.92 ± 0.08^****^ | 0.73 ± 0.09^##^ | 0.67 ± 0.25 | 36.486 | ＜0.0001 |
| 48 h | 0.63 ± 0.10 | 0.62 ± 0.12 | 0.57 ± 0.07^####^ | 0.73 ± 0.19 | 15.733 | ＜0.0001 |
| 72 h | 0.67 ± 0.02 | 0.83 ± 0.08 | 0.72 ± 0.18 | 0.74 ± 0.13 | 27.73 | 0.117 |
| Total | 0.20 ± 0.06 | 0.90 ± 0.10 | 0.68 ± 0.13 | 0.71 ± 0.20 | 2.303^a^ | ＜0.001^a^ |
| *F* | 10.977 | 1.182 | 2.981 | 1.407 | 6.996^b^ | ＜0.001^b^ |
| *P* | ＜ 0.001 | 0.32 | 0.066 | 0.261 |  |  |

a, the F-statistic and P-value for the main effect. b, the F-statistic and *P*-value for the interaction effect. Compared to the Sham group, * *P* < 0.05, ** *P* < 0.01, *** *P* < 0.001, **** *P* < 0.0001; compared to the CLP group, # *P* < 0.05, ## *P* < 0.01, ### *P* < 0.001, #### *P* < 0.0001.

**Table S11. The effect of emodin on MALT1 protein levels in septic mice**

| Time | Group | | | Total | F | *P* |
| --- | --- | --- | --- | --- | --- | --- |
|  | Sham | CLP | Emodin |  |  |  |
| 24 h | 0.31 ± 0.08 | 1.22 ± 0.15^****^ | 0.79 ± 0.11^##^ | 0.77 ± 0.38 | 27.935 | ＜0.0001 |
| 48 h | 0.38 ± 0.07 | 1.15 ± 0.13^****^ | 0.65 ± 0.09^####^ | 0.73 ± 0.31 | 21.446 | ＜0.0001 |
| 72 h | 0.52 ± 0.10 | 0.94 ± 0.12^**^ | 0.71 ± 0.08^#^ | 0.74 ± 0.13 | 14.882 | 0.001 |
| Total | 0.20 ± 0.06 | 0.90 ± 0.10 | 0.68 ± 0.13 | 0.71 ± 0.20 | 2.303^a^ | ＜0.001^a^ |
| *F* | 0.25 ± 0.06 | 1.08 ± 0.09 | 0.68 ± 0.12 | 4.217 | 30.172^b^ | ＜0.0001^b^ |
| *P* | 0.032 | <0.001 | 0.046 | 0.032 |  |  |

a, the F-statistic and P-value for the main effect. b, the F-statistic and *P*-value for the interaction effect. Compared to the Sham group, * *P* < 0.05, ** *P* < 0.01, *** *P* < 0.001, **** *P* < 0.0001; compared to the CLP group, # *P* < 0.05, ## *P* < 0.01, ### *P* < 0.001, #### *P* < 0.0001.


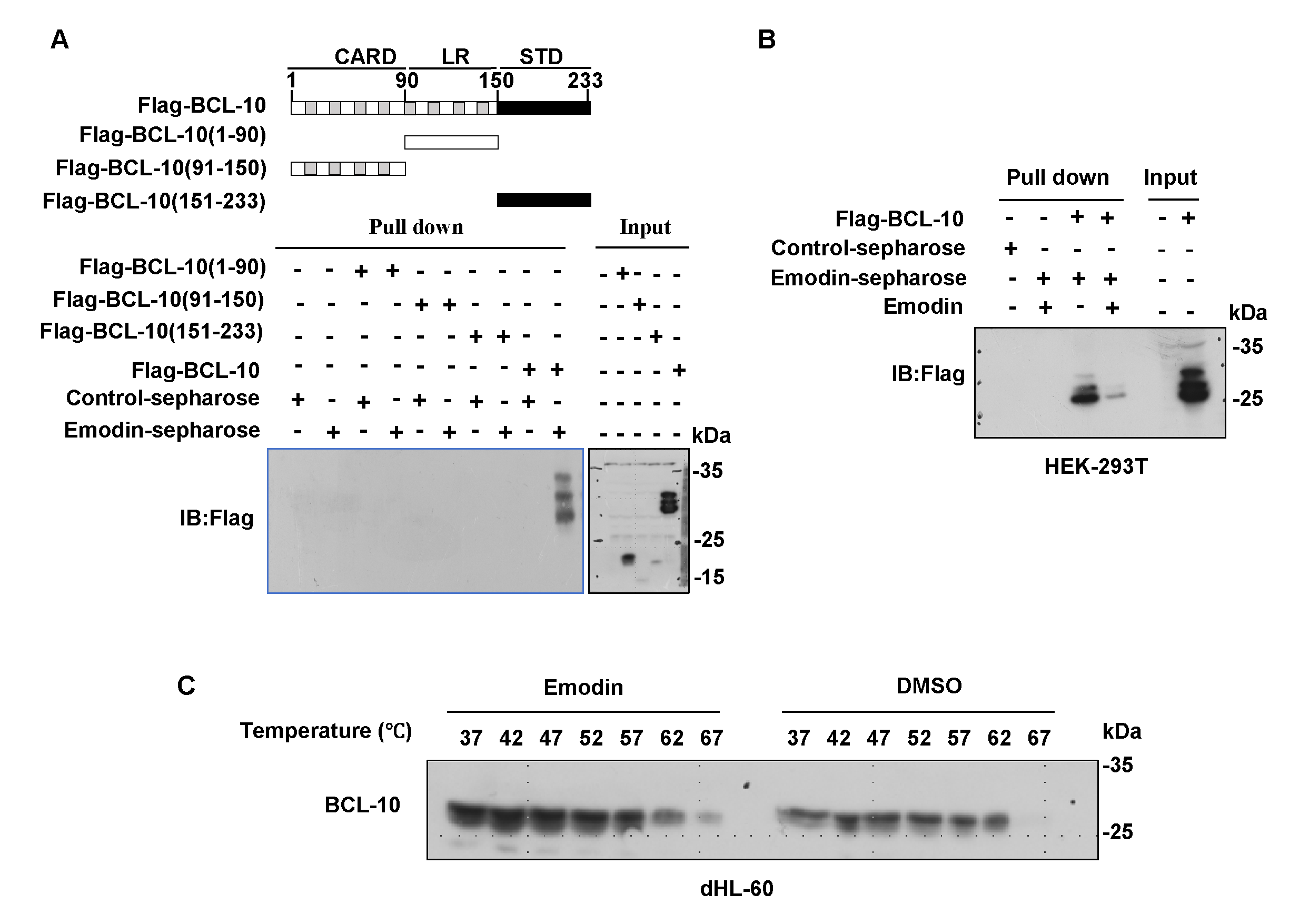


**Figure S5. Emodin directly and specifically binds to BCL-10.** A. Validation of direct binding between emodin and BCL-10 in cell lysates by pull-down assay. B. Verification of competitive binding of free emodin to the same site on BCL-10. C. Confirmation of emodin-induced conformational stabilization of BCL-10 through thermal shift assay.


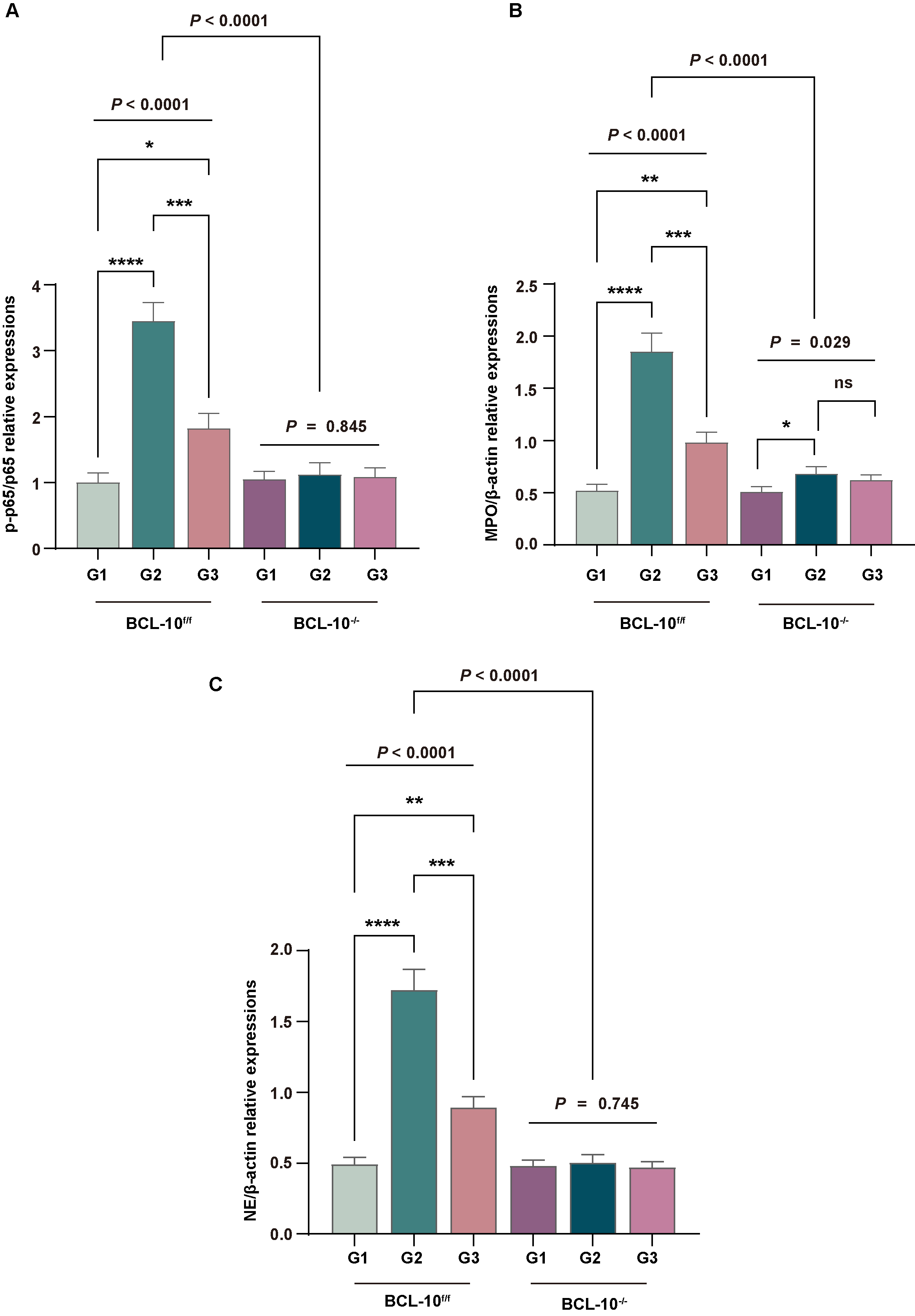


**Figure S6. Western blot analysis of p-p65 (A), MPO (B), and NE (C) expression in BCL-10 ^f/f^ and BCL-10 ^-/-^mice under Sham, CLP, and Emodin intervention.** **P* < 0.05, ***P* < 0.01, ****P* < 0.001, ****P* < 0.0001; n.s., no significant difference (*P* > 0.05)*.* G1, G2, and G3 represent the sham group, CLP model group, and Emodin intervention group, respectively.
